# Potent neutralizing RBD‐specific antibody cocktail against SARS‐CoV‐2 and its mutant

**DOI:** 10.1002/mco2.79

**Published:** 2021-06-24

**Authors:** Lina Jia, Yan‐Ping Liu, Li‐Fei Tian, Chao Xiong, Xin Xu, Honge Qu, Weixi Xiong, Dong Zhou, Feng Wang, Zheng Liu, Xiao‐Xue Yan, Wenqing Xu, Lin Tang

**Affiliations:** ^1^ Department of Neurology State Key Lab of Biotherapy and Cancer center West China Hospital Sichuan University and Collaborative Innovation Center for Biotherapy Chengdu Sichuan China; ^2^ National Laboratory of Biomacromolecules Chinese Academy of Sciences (CAS) Center for Excellence in Biomacromolecules Institute of Biophysics, Chinese Academy of Sciences Beijing China; ^3^ Wuxi Biortus Biosciences Co. Ltd. Jiangyin China; ^4^ School of life and health Kobilka Institute of Innovative Drug Discovery the Chinese University of Hong Kong Shenzhen China; ^5^ Shanghai Institute for Advanced Immunochemical Studies and School of Life Science and Technology ShanghaiTech University Shanghai China

**Keywords:** cocktail, Cyro‐EM, epitopes group, neutralizing antibody, receptor‐binding domain, SARS‐CoV‐2

## Abstract

The ongoing pandemic caused by severe acute respiratory syndrome coronavirus 2 (SARS‐CoV‐2) and its variants has posed a serious global public health emergency. Therapeutic interventions or vaccines are urgently needed to treat and prevent the further dissemination of this contagious virus. This study described the identification of neutralizing receptor‐binding domain (RBD)‐specific antibodies from mice through vaccination with a recombinant SARS‐CoV‐2 RBD. RBD‐targeted monoclonal antibodies (mAbs) with distinct function and epitope recognition were selected to understand SARS‐CoV‐2 neutralization. High‐affinity RBD‐specific antibodies exhibited high potency in neutralizing both live and pseudotype SARS‐CoV‐2 viruses and the SARS‐CoV‐2 pseudovirus particle containing the spike protein S‐RBD_V367F_ mutant (SARS‐CoV‐2(V367F)). These results demonstrated that these antibodies recognize four distinct groups (I–IV) of epitopes on the RBD and that mAbs targeting group I epitope can be used in combination with mAbs recognizing groups II and/or IV epitope to make mAb cocktails against SARS‐CoV‐2 and its mutants. Moreover, structural characterization reveals that groups I, III, and IV epitopes are closely located to an RBD hotspot. The identification of RBD‐specific antibodies and cocktails may provide an effective therapeutic and prophylactic intervention against SARS‐CoV‐2 and its isolates.

## Graphical abstract

- The neutralizing receptor‐binding domain‐specific antibodies from mice showed high affinity against SARS‐CoV‐2 live and pseudovirus particle bearing the wildtype and SV367F mutant protein.
- The antibodies selected for distinct epitopes on the RBD have different levels of competition capacity with ACE2 binding to RBD.
- The combination of them in usage could provide an effective way to control SARS‐CoV‐2 transmission.

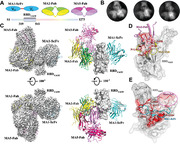

## 1 INTRODUCTION

The novel coronavirus SARS‐CoV‐2 caused Coronavirus disease 2019 (COVID‐19), which belongs to the *Betacoronavirus* genus and shares high sequence homology with SARS‐CoV (82%).^1^ The emergence of SARS‐CoV‐2 posed a serious global health emergency; it spread rapidly worldwide, leading to a COVID‐19 pandemic that infected more than 79 million people and killed over 1.7 million. Several studies have shown the protective roles of neutralizing antibodies against SARS‐CoV‐2. Plasma from convalescent individuals, which contains neutralizing antibodies, inhibited virus infection and has a potential for therapeutic interventions.^2^, ^3^ Given the limitations of plasma for therapeutic use, several research groups have identified neutralizing antibody candidates from humanized mice and convalescent individuals.^4^, ^5^, ^6^, ^7^, ^8^, ^9^, ^10^, ^11^

SARS‐CoV‐2 and SARS‐CoV infect host cells in a similar mechanism. These viruses apply angiotensin‐converting enzyme 2 (ACE2) as a cell receptor for viral entry through their transmembrane spike glycoprotein (S).^12^, ^13^, ^14^, ^15^, ^16^, ^17^ The SARS‐CoV‐2 S protein have two functional subunits: an N‐terminal S1 domain and a C‐terminal S2 domain (S2 domain protruding from the viral surface). RBD domain of the S1 tightly binds to the peptidase domain of ACE2, playing key roles in determining host range, tropism, and infectivity. The coronavirus RBD is the main target of neutralizing antibodies and has been a focus of vaccine design and therapeutic efforts.^18^, ^19^, ^20^, ^21^ SARS‐CoV and the Middle East respiratory syndrome coronavirus RBD‐based antibodies have shown neutralization activities in the previous studies.^22^ The SARS‐CoV‐2 RBD shares 50% sequence identity with that of SARS‐CoV, explaining why formerly SARS‐CoV antibodies could not neutralize SARS‐CoV‐2.^23^ The neutralizing anti‐RBD monoclonal antibodies (mAbs) are believed to disrupt the virus–receptor engagement. Several potent neutralizing antibodies from convalescent patients, which recognize the SARS‐CoV‐2 RBD, have been recently reported.^5^, ^6^, ^7^, ^8^, ^9^, ^10^, ^11^ This study described the identification of potent neutralizing RBD‐specific mAbs from mice vaccinated by a recombinant SARS‐CoV‐2 RBD. These antibodies exhibited a higher binding affinity to the RBD and high potency to neutralize both live and pseudotype SARS‐CoV‐2 viruses and SARS‐CoV‐2(V367F) pseudovirus. Overall, these antibodies recognize four distinct epitopes on the RBD, and cocktails containing mAbs targeting different antigenic sites showed higher potency to neutralize the SARS‐CoV‐2 virus and SARS‐CoV‐2(V367F) pseudovirus particle.

## 2 RESULTS

### 2.1 Characterization of high‐affinity RBD‐specific mAbs

**FIGURE 1:**
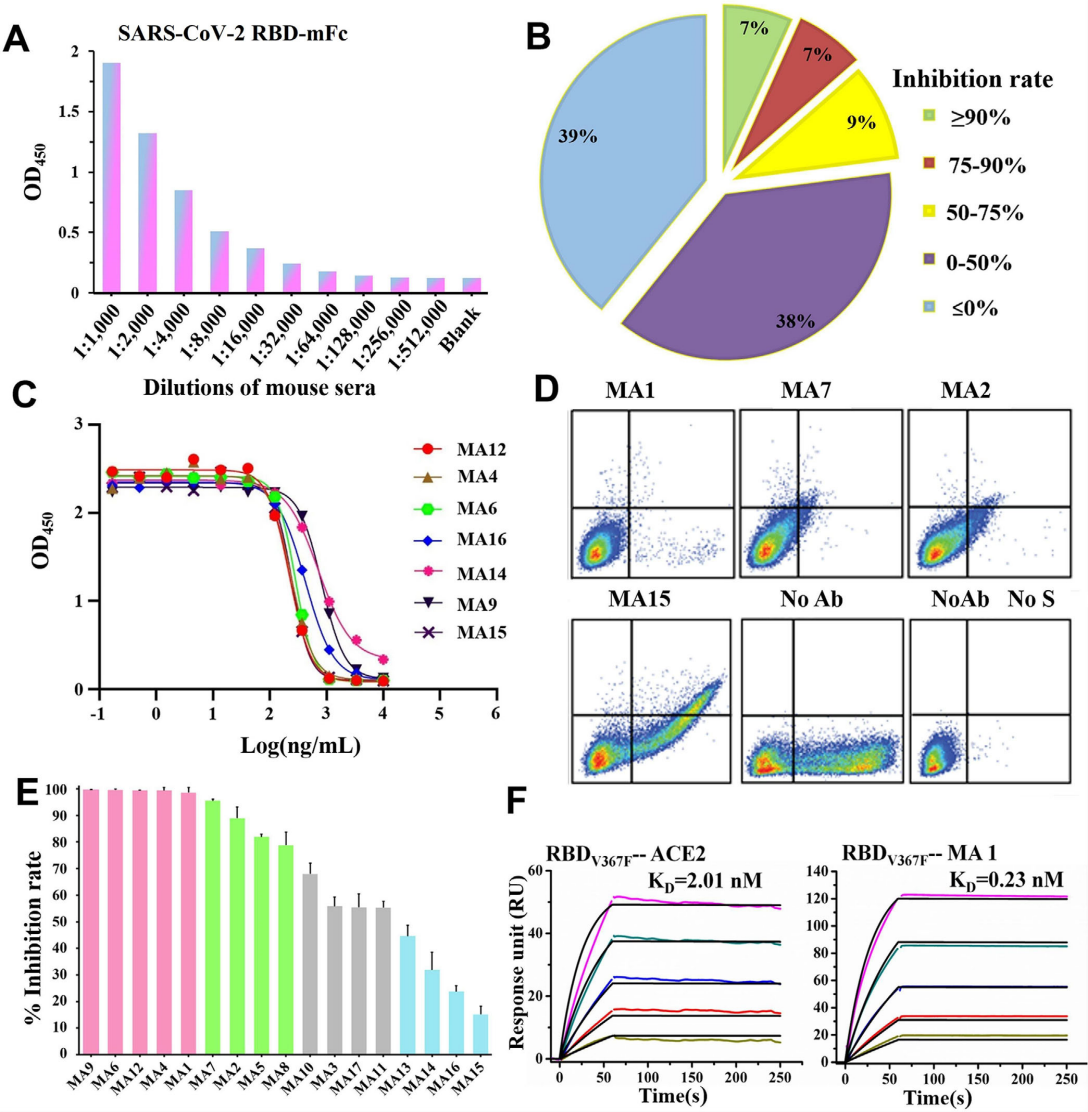
Characterization of RBD‐specific mAbs inhibiting binding of the S to ACE2 receptor. (A) Serological antibody responses to the SARS‐CoV‐2 RBD‐mFc evaluated by ELISA. (B) Diagram shows the percentage of hybridoma clones with different competition levels against ACE2 binding to the SARS‐CoV‐2 RBD. Inhibition rate ≦ 0% indicated approximately 39% of total clones could not compete with ACE2. (C) Inhibitory curves of representative mAbs competing with ACE2. (D) Representative mAbs block SARS‐CoV‐2 S binding to ACE2 in FACS‐based assay. (E) The inhibition rate of all selected mAbs was measured by flow cytometry with 10 μg/ml of each antibody, and experiments were performed three times. (F) Binding kinetics of MA1 and ACE2 with SARS‐CoV‐2 RBD_V367F_. The purified soluble SARS‐CoV‐2 RBD_V367F_ was covalently immobilized onto a CM5 sensor chip followed by injection of ACE2 or MA1 with five different concentrations. The black line indicates the experimentally derived curves, and the colored lines represent the fitted curves based on the experimental data

As the RBD mediates entry into host cells through direct interaction with ACE2, and it is the prime target of neutralizing mAbs, a recombinant RBD‐Fc was used to immunize mice to isolate RBD‐targeted mAbs (Figures 1A and S1.) Using enzyme‐linked immunosorbent assay (ELISA) competition and surface plasmon resonance (SPR) assays, 17 mAbs binding to the SARS‐CoV‐2 RBD were identified (Figures 1B, 1C, and S1‐S3).

To identify mAbs that inhibit the interaction between the SARS‐CoV‐2 RBD and ACE2, 74 of 220 positive clones were finally selected for a competition assay in the presence of ACE2 for binding to the SARS‐CoV‐2 RBD (Table S1). Specifically, the supernatant of the 74 clones was added to an ELISA plate coated with a recombinant human ACE2 in the presence of a His‐tagged SARS‐CoV‐2 RBD. The RBD binding to ACE2 was visualized by anti‐His secondary antibodies at an optical density of 450 nm. The competing power of each antibody clone was measured via the percent reduction in RBD binding with the ACE2 receptor. As shown in Figure 1B and Table S1, approximately 23% of total clones were competitive against ACE2. Seventeen antibody clones that reduced ACE2 binding by more than 50% were selected; their light and heavy chains were cloned to the linear expression cassettes. These mAbs were produced by either using a hybridoma cell line or transfection of linear expression cassettes, and their competing potency with ACE2 binding to the SARS‐CoV‐2 RBD was analyzed. Ten mAbs demonstrated a higher level of competition activity, with half‐maximal inhibitory concentrations (IC_50_) values ranging from 1.24 to 3.14 nM, and the other seven mAbs had only slightly lower competing power, with variable IC_50_ values ranging from 3.25 to 11.13 nM (Figures 1C, S1C, and S1D, and Table S2). To differentiate the levels of competition among these mAbs, flow cytometry analysis was used to define whether the 17 antibodies can effectively inhibit the binding of the S to the ACE2 expression on the surface of HEK293T cells. As shown in Figures 1D, 1E, and S4, five mAbs (MA1, MA4, MA6, MA9, and MA12) completely blocked recombinant S‐His from binding to cell‐associated ACE2, and five mAbs (MA2, MA5, MA7, MA8, and MA10) also strongly interfered with ACE2 binding. These results demonstrated that 10 of the 17 mAbs have higher competition levels against ACE2 for binding to the SARS‐CoV‐2 S protein, suggesting that these mAbs would potentially neutralize SARS‐CoV‐2.

The binding activity of these mAbs to the SARS‐CoV‐2 RBD was further analyzed using ELISA and SPR (Figures S2 and S3), and the K_D_ (dissociation constants) ranged from 10^–10^ to 10^–9^ M. These data showed that six mAbs (MA1–MA4, MA6, and MA7) have a higher binding affinity to the SARS‐CoV‐2 RBD with subnanomolar K_D_ values than the rest mAbs in this study. The 10 aforementioned mAbs with higher competition levels against ACE2 strongly bound to the SARS‐CoV‐2 RBD with a higher binding affinity than most other mAbs. Similarly, most of the 10 mAbs with higher competition levels against ACE2 and higher binding affinity to the SARS‐CoV‐2 RBD exhibited greater binding affinity to the SARS‐CoV‐2 RBD_V367F_. Interestingly, the binding affinity of RBD_V367F_ to ACE2 (Figure 1F) was increased by twofold than that of the wild‐type RBD to ACE2, which correspond to a previous study.^14^ MA1, one of the most competitive mAbs, demonstrated a 10‐fold higher binding affinity to the SARS‐CoV‐2 RBD_V367F_ than ACE2 (Figures 1F and S5). These results collectively suggested that the top 10 mAbs with high avidity would be potential candidates for neutralizing SARS‐CoV‐2 and its mutants.

### 2.2 Neutralization properties of RBD‐specific mAbs

**FIGURE 2:**
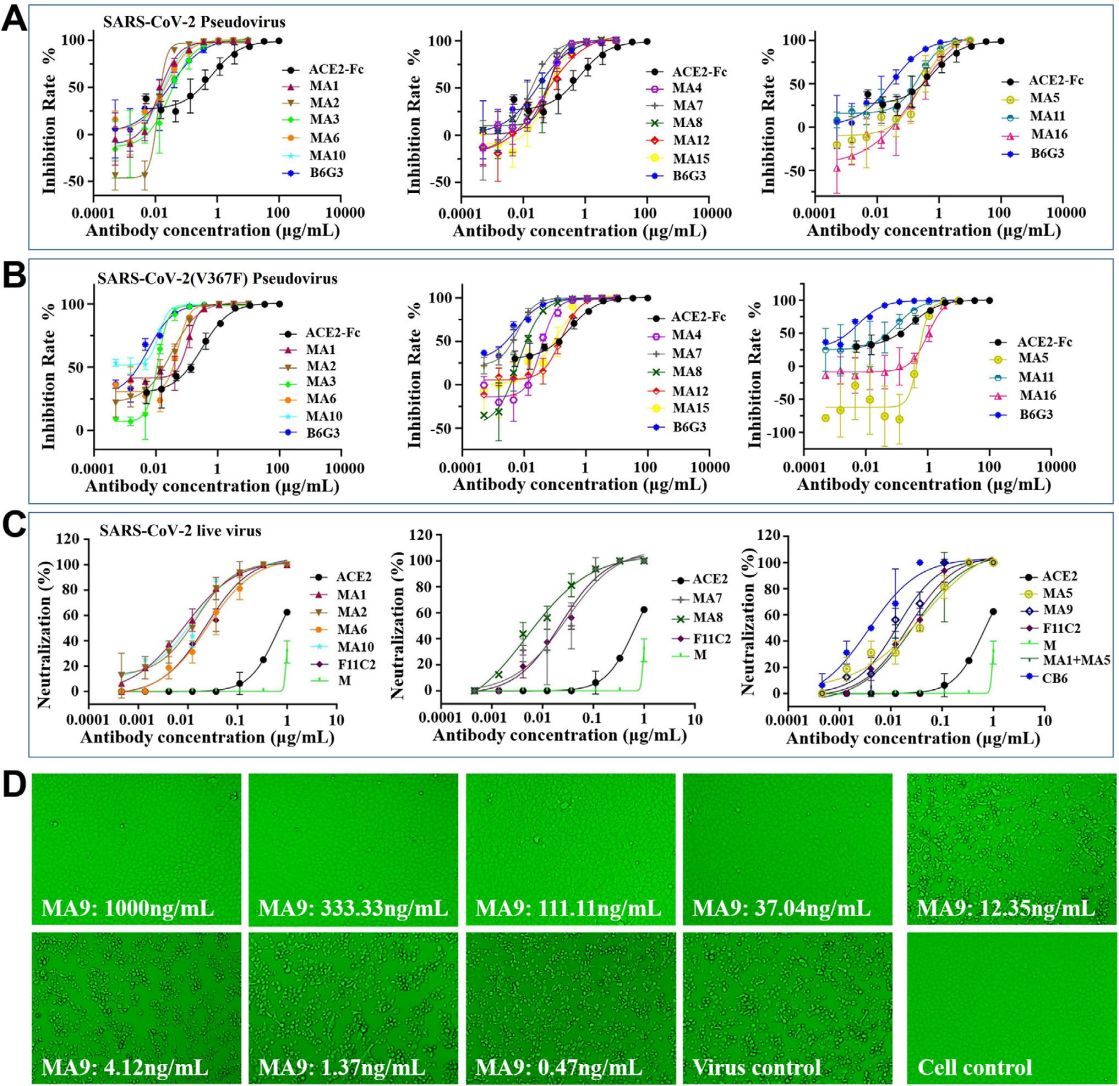
Neutralization of the SARS‐CoV‐2 pseudovirus and live virus and the SARS‐CoV‐2(V367F) pseudovirus by mAbs. (A and B) SARS‐CoV‐2 and SARS‐CoV‐2(V367F) pseudovirus were incubated with threefold serially diluted mAbs. The mixtures were then added to Huh7 cells. After 24 h incubation, neutralization potencies of mAbs were evaluated in a luciferase assay system. (C) The mixture of live SARS‐CoV‐2 virus and serially diluted mAbs was added to Vero‐E6 cells. After 72 h incubation, ND_50_ was calculated by fitting the CPE proportion with serially diluted antibody to a sigmoidal dose‐response curve. (D) CPE for representative MA9 was observed daily and recorded on day 3 post‐exposure

Whether high‐affinity RBD‐targeted mAbs could efficiently neutralize SARS‐CoV‐2 live and pseudovirus and SARS‐CoV‐2(V367F) pseudovirus particles was determined. The majority (MA1–MA4 and MA6–10) of the mAbs showed high neutralizing activity against the SARS‐CoV‐2 pseudovirus and live virus. MA1–MA3, MA6, and MA10 mAbs were the most potent neutralizers among the total mAbs evaluated, with IC_50_ values in the subnanomolar range (Figure 2 and Table S2). A similar neutralizing potency of most mAbs against the SARS‐CoV‐2(V367F) pseudovirus particle was observed. However, MA1 was less potent to neutralize the SARS‐CoV‐2(V367F) pseudovirus than the wild‐type SARS‐CoV‐2 and live virus, indicating that a conformational change of RBD_V367F_ might have occurred within these antigenic sites. Based on the binding data, MA5 strongly interacted with the SARS‐CoV‐2 RBD and inhibited binding of the RBD to ACE2; however, it was less potent to neutralize the SARS‐CoV‐2 pseudovirus, live virus, and SARS‐CoV‐2(V367F) pseudovirus compared with other mAbs, indicating that it might have recognized a distinct epitope on the RBD, limiting its neutralizing activity.

### 2.3 Epitope specificity determined by a binding competition assay

RBD‐Fc and dithiothreitol (DTT)‐reduced RBD‐Fc were immobilized on ELISA plates to determine whether these antibodies recognize a conformational epitope. All 17 monodonal antibodies responded to natural RBD‐Fc, but not to DTT‐reduced RBD‐Fc, indicating that they recognized the disulfide bond‐dependent conformational epitopes expressed on the S protein RBD.

**FIGURE 3:**
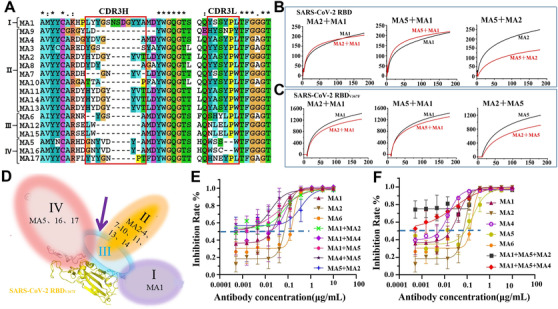
Four distinct antigenic sites of the RBD targeted by four groups of mAbs, which can make potent neutralizing cocktails against SARS‐CoV‐2 and SARS‐CoV‐2(V367F). (A) The CDR3 sequence comparison between 17 mAbs defines four different groups of mAbs. (B) The sensorgrams showed that MA1 does not compete with MA2 or MA5 for binding to the SARS‐CoV‐2 RBD, whereas MA2 competes with MA5 for binding to the RBD. (C) The sensorgrams showed that MA1 does not compete with MA2 or MA5 for binding to the SARS‐CoV‐2 RBD_V367F_, and MA2 does not compete with MA5 for binding to the RBD. Pairs of testing antibodies were sequentially applied to the covalently immobilized RBD on a CM5 sensor chip, and the level of reduction in RU compared with or without prior antibody incubation is the key criterion to determine two mAbs that recognize separate or closely situated epitopes. (D) Models of four distinct antigenic sites on the RBD. Group III epitope overlaps with three other groups, whereas group I is separated from groups II and IV. (E and F) Triple‐mAb and pair‐mAb cocktails with threefold serial dilution were incubated with SARS‐CoV‐2(V367F) pseudovirus. The mixtures were then added to Hu7 cells, and the neutralization potencies of mAb cocktails were evaluated in a luciferase assay system

To understand the fine specificity of mAbs, an ACE2 competition assay was developed using one biotinylated mAb as the reference competing with other antibodies for binding to the SARS‐CoV‐2 RBD. For instance, MA9 was first biotinylated, and the inhibitory roles of other mAbs on MA9 binding to the RBD was measured. Thirteen mAbs (MA) competed with the biotinylated MA9, but the others did not block MA9 from binding to the RBD (Figure S6). A similar competing pattern was observed when MA2–MA4, MA6–MA8, MA10, MA11, MA13, and MA14 were biotinylated, indicating that they all competed for a similar conformational epitope on the RBD. By contrast, when MA1, one of the most potent neutralizing Abs, was biotinylated, only four mAbs (MA9, MA6, MA12, and MA15) were shown to compete with MA1 for binding to the RBD. Therefore, MA1 may recognize a distinct epitope from the other mAbs and is regarded under group I mAb (Figure 3D). MA6, MA12, and MA15 competed with every biotinylated mAb; thus, they comprised a distinct group called group III mAb. The other MA9 competing antibodies shared a similar competition pattern; therefore, they recognized an overlapping epitope on the RBD, forming group II mAbs (Figure 3D). Three MA9 noncompetitive mAbs were further biotinylated and tested in a similar ACE2 competition assay. MA16, MA17, and MA5 competed only with mAbs within group II, suggesting that they formed a separate group defined here as group IV mAbs (Figure 3D). The four groups with special epitopes were in accordance with the CDR3 sequence comparison between 17 mAbs (Figure 3A).

The finding that group III mAbs (MA6, MA12, and MA15) were competitive with almost all other mAbs in the ACE2 competition assay suggested that they possibly recognize an essential neutralizing epitope on the RBD, overlapping with the remaining epitopes defined in this study. This finding was confirmed using SPR for the selected representative mAbs from each group for a competition assay in a pairwise fashion (Figures 3B and S7). MA6 and MA12 competed with the rest of the mAbs for binding to the immobilized RBD, consistent with the ELISA competition data. Instead, MA1 and MA5 were found to weakly compete with group III (MA6 and MA12) mAbs but showed no competition between them, indicating that their epitopes on RBD are separated from each other. One notable feature of MA1 (group I) mAb is that it does not compete with both group II (MA2, MA7, and MA10) and group IV (MA5) mAbs for the recognition of the SARS‐CoV‐2 RBD. The epitope specificities of the selected mAbs on the RBD_V367F_ mutant were also investigated, and a similar competition pattern was observed among mAbs (Figure 3C). MA5 (group IV) mAb weakly competed with MA2 and MA10 (group II) for binding to the RBD; however, this competition did not exist for the RBD_V367F_ mutant (Figure S8), indicating that mAbs from groups II and IV can simultaneously bind to RBD_V367F_. Additionally, a triple‐mAb complex of RBD_V367F_ with MA1scFv, MA2Fab, and MA5Fab (Figure S9) and a pair of antibody complex of RBD with MA1scFv and MA2Fab (Figure S10) were successfully assembled. As group I MA1, group II MA2 to MA4, and group IV MA5 demonstrated a potent neutralizing activity toward SARS‐CoV‐2 pseudoviruses, antibody cocktails comprising mAbs from group I and those from groups II and/or IV could be used potentially for therapeutic interventions against SARS‐CoV‐2 and its mutants.

### 2.4 RBD‐specific mAb cocktails have potent neutralizing activity against SARS‐CoV‐2(V367F) pseudovirus

To determine whether mAbs from groups I, III, and IV can be used together for synergistic enhancement in their neutralization potency, MA1 (group I), MA2 (group III), MA4 (group III), MA5 (group IV), and MA6 (group II) were chosen for the pseudovirus neutralization assay. As shown in Figures 3E and 3F, the triple‐mAb cocktail containing MA1 and MA5 combined with either MA2 or MA4 exhibited a higher neutralization potency than the pair‐mAb cocktails or single usage of mAb against SARS‐CoV‐2(V367F) pseudovirus. In line with the isolation of a three‐mAb RBD complex, these findings confirmed that mAbs recognizing sites I, III, and IV could bind to RBD_V367F_ simultaneously, leading to an increase in neutralization activity against SARS‐CoV‐2(V367F). Generally, the pair‐mAb cocktails had a higher level of neutralizing activity than a single mAb from each group. These results suggested that antigenic site I is set apart from site IV than site II, and the close distance between sites II and IV in the wild‐type RBD could limit the simultaneous access of antibodies targeting these two sites. The three‐mAb binding to RBD_V367F_ might have resulted from conformational changes that occurred within sites II and IV, and the distance between them was somehow more separate in the RBD_V367F_ mutant. This was further supported by following the structural determination of the MA1ScFv/MA2Fab/MA5Fab/ RBD_V367F_ complex.

### 2.5 Structural characterization of mAbs targeting sites I, II, and IV

**FIGURE 4:**
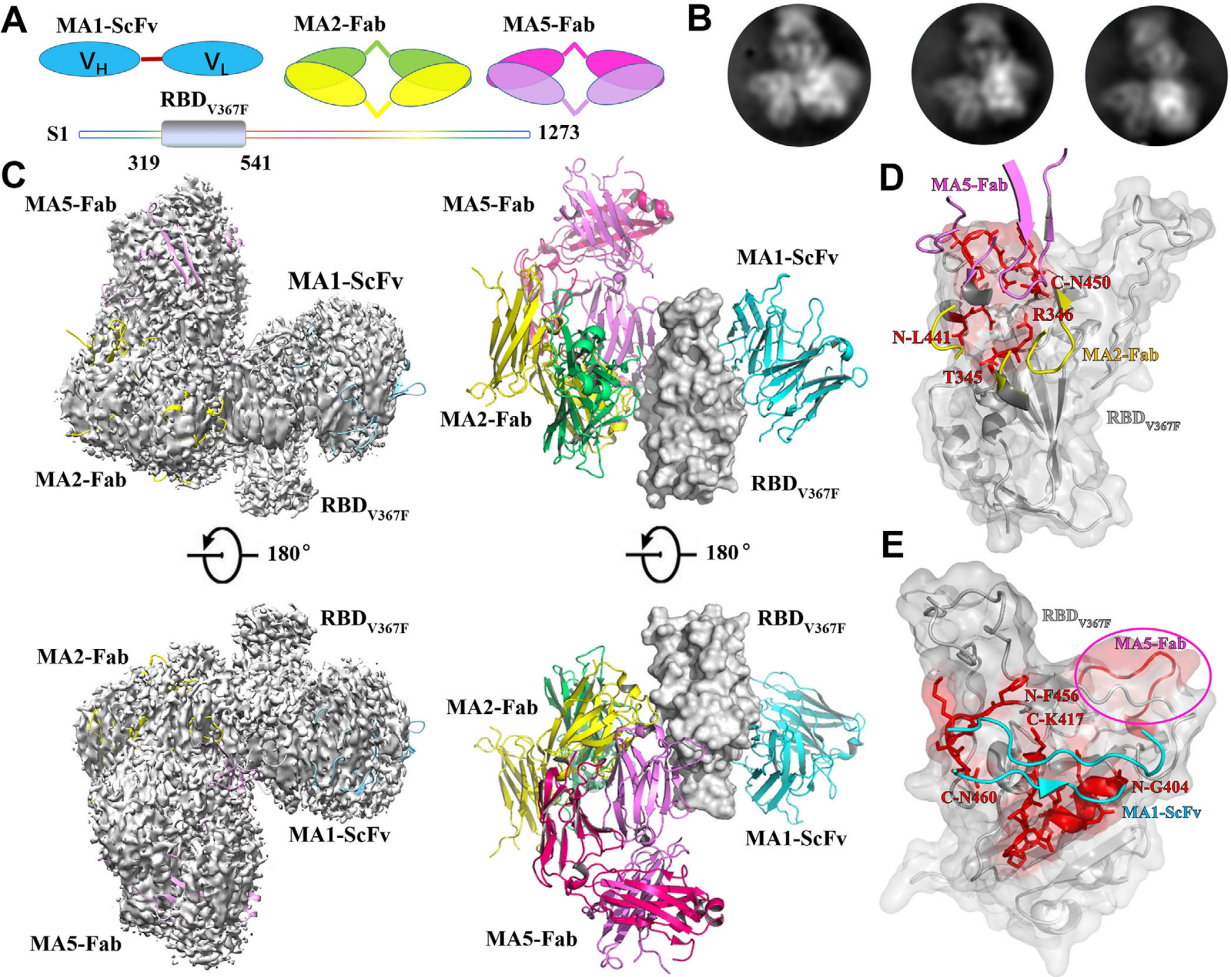
Structural basis for the recognition of the RBD_V367F_ by mAbs cocktails containing MA1ScFv, MA2Fab, and MA5Fab. (A) Schematic of MA1ScFv, MA2Fab, MA5Fab, and SARS‐CoV‐2 RBD_V367F_ are marked in sky blue, forest green, yellow, rosy, purple, and silver, respectively. (B) Representative 2D class averages of the RBD_V367F_ bound to MA1ScFv, MA2Fab, and MA5Fab. (C) Cryo‐electron microscopy density map of the the RBD_V367F_ in complex with MA1ScFv, MA2Fab, and MA5Fab. Details are indicated, and the color scheme is the same as in (A). (D and E) Details of the interface between the antibodys and SARS‐CoV‐2 RBD_V367F_. The binding hotspot involved in interaction of MA1, MA2, and MA5 epitopes is labeled with red. And, MA5 binding site, including residues L441‐N450 of RBD is emphasized by transparent purple circle

SARS‐CoV‐2 neutralization, mAbs recognizing sites I, II, and IV were selected for the structural characterization of their variable domains in complex with the RBD by cryo‐electron microscopy (cryo‐EM). MA1scFv was transiently expressed in HEK293 cells and further purified by size exclusion chromatography, and the Fab fragments of MA5 and MA7 were prepared in a similar way as described previously.^14^ The three‐dimensional reconstructions of MA1ScFv/MA2Fab/MA5Fab/RBD_V367F_ complex were determined (Figures 4A‐4C). And the RBD density was not well resolved in MA1ScFv/MA2Fab/RBD complex because of its flexibility and the preferred particle orientation on the EM grid (Figure S11).

All three antibodies recognized epitopes on the same side of the RBD and surrounded the RBD like a shamrock (Figure 4C). A direct comparison between MA1ScFv/MA2Fab/RBD and MA1ScFv/MA2Fab/MA5Fab/RBD_V367F_ complex defines the relative orientation and position of MA1ScFv and MA2Fab, and MA5Fab may be located near MA1 in MA1ScFv/MA2Fab/MA5Fab/RBD_V367F_ complex. The three MAbs of the cocktail can simultaneously bind to distinct regions of the RBD; MA5 and MA2 recognizing a more conserved patch across SARS‐CoV‐2 RBD (residues 441–450), corresponding to an ACE2‐interactional region (Figure 4D). And the the epitopes of SARS‐CoV‐2 revealed by MA1 (residues 456–460) suggested that antibodies targeting the RBD primarily are more likely to be ACE2‐specific binding site (Figure 4E).

Structural analysis revealed that the antigenic sites I, II, and IV were located to a closely related RBD region, which may partially overlap with the ACE2 binding site. This finding agreed with that in a flow cytometry competition assay that antibodies targeting sites I, III, and IV have different levels of competition capacity with ACE2 binding to RBD. Based on a recent study,^9^ the neutralization potency of antibodies correlated with their hindrance with ACE2 binding. The antibodies selected for distinct epitopes on the RBD could inhibit viral attachment to the host cell differently, and their combination in usage could provide an effective way to control SARS‐CoV‐2 transmission.

## 3 DISCUSSION

The SARS‐CoV‐2 S glycoprotein is the prime antigen inducing immune responses during infection, and S‐based vaccines enter into phase III clinical trials.^24^, ^25^ One drawback of the full‐length coronavirus S‐based vaccine is that it contains undesirable antigenic sites, resulting in antibodies that do not neutralize virus infection.^18^ However, the RBD domain of the S protein is responsible for recognizing the host cell receptor and is the major target of 90% of the neutralizing antibodies. Recent studies have demonstrated that RBD‐directed mAbs isolated from convalescent COVID‐19 donors efficiently neutralize SARS‐CoV‐2, whereas non‐RBD antibodies fail to exhibit pseudovirus neutralizing capability.^5^, ^18^ Furthermore, the US Food and Drug Administration urgently approved a cocktail containing RBD‐specific mAbs for clinical usage to treat patients with COVID‐19. This study identified 10 potent neutralizing RBD‐specific mAbs targeting four distinct antigenic sites (sites I–IV). An ACE2 competition assay was developed to screen out antibodies with low competition potency against ACE2; therefore, most of the selected RBD‐specific mAbs exhibited a high level of competition with ACE2 binding and high binding affinity to the RBD. The competitive ability of ACE2 is closely related to the neutralizing potency of RBD‐specific mAbs; this correlation was used as a strategy for the identification of highly potent mAbs by several research groups.^4^, ^5^, ^6^, ^8^ These data demonstrated that most mAbs neutralize both live and pseudotype SARS‐CoV‐2 viruses and the SARS‐CoV‐2(V367F) pseudovirus particle with IC_50_ values in the subnanomolar range. These results highlighted the importance of using both functional and binding tests to assess the neutralizing potencies of mAbs.

The SARS‐CoV‐2 RBD contains at least four different but overlapping antigenic sites recognized by mAbs with different levels of neutralization potencies. Cocktails containing mAbs recognizing different epitopes demonstrated a higher neutralization potency against SARS‐CoV‐2. Site I‐targeted mAb (group I) can be used together with mAbs from group II or IV to develop mAb cocktails against SARS‐CoV‐2. Group II mAbs recognize epitopes that slightly overlap with the epitopes recognized by group IV mAbs; however, these two epitopes become separate from each other within the RBD_V367F_ because of conformational changes. A triple‐mAb cocktail (MA1–MA2–MA5) was developed against the SARS‐CoV‐2(V367F) isolate. By contrast, group III mAbs shared an overlapping binding site with groups I, II, and IV mAbs; thus, these mAbs can only be used singly to neutralize SARS‐CoV‐2.

Cryo‐EM analysis showed that MA1 mAb (group I) along with mAbs from group II or IV bound to the RBD simultaneously, whereas MA1 (group I) mAb, MA2 (site III), and MA5 (site IV) bound to the RBD_V367F_ together. These structural data reveal that antigenic site I is distinct from sites II and IV; thus, antibodies targeting these sites can make cocktails for therapeutic use.

In conclusion, 10 potent neutralizing mAbs against SARS‐CoV‐2 with higher binding affinity to the RBD were identified. The majority of the mAbs neutralize the SARS‐CoV‐2(V367F) pseudovirus equally well, with group II mAbs, such as MA7 and MA8, exhibiting a remarkable neutralizing potency, with IC_50_ of about 42 pM. This finding suggested that group II mAbs prefers the conformation of the RBD_V367F_. Furthermore, this study structurally and functionally defined that group I antibodies could be used along with group II or IV antibodies to develop pair‐antibody cocktails against SARS‐CoV‐2 and its mutant. Meanwhile, groups I, II, and IV mAbs can be used to prepare triple‐mAb cocktails against the SARS‐CoV‐2(V367F) isolate. SARS‐CoV‐2 is still evolving and continuing to circulate in humans. Further studies are required to address whether the selected RBD‐specific mAbs can neutralize other SARS‐CoV‐2 isolates. We have identified the potent neutralizing mAbs maybe provide an effective prophylactic and therapeutic intervention against the continual dissemination of COVID‐19.

## 4 MATERIALS AND METHODS

### 4.1 Recombinant expression

Recombinant RBDs and trimeric S for SARS‐CoV‐2 and the human ACE2 N‐terminal domain (19‐615AA) were expressed using either the insect baculoviru (Invitrogen) or transient transfection of HEK293 cells. SARS‐CoV‐2 RBD (residues Arg319‐Phe541) or its mutant RBD_V367F_ was cloned into pFastBac vectors with a gp67 secretion signal at the N‐terminus and 6xHis tag at the C‐terminus. After infecting Hi5 cells with the high‐titer virus, recombinant RBD and S protein were secreted into the medium. Affinity purification and size exclusion chromatography were used for the purification of RBDs and S protein. The purification procedures of RBDs and S protein from mammalian cells were almost the same, except that HEK293 cells were transiently transfected by expression vectors with the target genes.

### 4.2 ELISA analysis

To screen for hybridoma clones that compete with ACE binding, the ELISA plates were coated with human ACE2‐hFc, and the supernatant from hybridoma clone and His‐tagged RBD were applied. The binding was visualized using anti‐His secondary antibodies at an optical density of 450 nm. The inhibitory rate was calculated via the percent reduction of S binding to ACE2. To determine the IC_50_ values of each antibody that competed with ACE2 binding, the ELISA plates were coated with His‐tagged RBD, and serial dilutions of the antibody were applied in the presence of ACE2 as a competing agent.

### 4.3 Flow cytometry analysis

The activity of mAbs to block SARS‐CoV‐2 S and ACE2 binding was measured by FACS. The vector of full‐length human ACE2 transfected HEK293T and incubated at 37°C for 36 h. Each antibody was incubated with S protein for 30 min, and then, the mAb/S mixture was added to ACE2‐expressing 293‐cells. Cells were stained with anti‐human IgG FITC, mAb binding, and anti‐His S binding. Then, cells were analyzed using FACSalibur flow cytometer (CellQuest software).

### 4.4 Affinity and epitope mapping determination by SPR

The affinity and binding kinetics of mAbs to SARS‐CoV‐2 RBD and RBD_V367F_ were analyzed using Biacore 8K (GE Healthcare). Via amine groups, the recombinant SARS‐CoV‐2 RBD or SARS‐CoV‐2 RBD_V367F_ was covalently immobilized to the CM5 chip in 10 mM sodium acetate, pH 5.0 for a final response unit of ∼200. SPR assays were run at a flow rate of 30 μl/min in HEPES (20 mM HEPES [pH 8.0], 150 mM NaCl, and 0.05% Tween 20). The sensorgrams were fit in a 1:1 binding model with Biacore Evaluation Software (GE Healthcare). For epitope mapping, SARS‐CoV‐2 RBD or SARS‐CoV‐2 RBD_V367F_ was immobilized to a CM5 chip for a final RU of approximately 1500. The first mAbs were injected into the chip until reached a binding steady state, and the second antibodies were then injected for 180 s. Whether two antibodies recognized different epitopes can be determined by their binding ability.

### 4.5 Pseudovirus and live SARS‐CoV‐2 neutralization assays

Serial dilutions of purified mAbs or mAb cocktails were mixed with incubating pseudoviruses at 37°C for 1 h. Cells for SARS‐CoV‐2 or SARS‐CoV‐2 (V367F) pseudovirus (∼15,000 per well) were added to the virus–antibody mixture. The IC_50_ of the mAbs was measured by luciferase activity 48 h after exposure to the virus–mAbs mixture using GraphPad Prism 7 Software. Live SARS‐CoV‐2 was performed using the cytopathic effect assay. All experiments were performed in a Biosafety Level 3 facility.

### 4.6 Cryo‐EM data processing and model building

A total of 1,990,360 particles of the MA1ScFv/MA2Fab/MA5Fab/RBD_V367F_ complex were auto‐picked and extracted with a box size of 170 × 170 pixels from 2265 micrographs (Figure S12). A low picking threshold was used to include the good particles as more as possible, and the particles were extracted and binned for 2D classification. We selected 1,365,928 good particles after two rounds of 2D classification. Initial 3D models were generated from 2D class averages by RELION 3.0‐beta. The selected particles were subjected to 3D classification. Four classes, 1,221,238 particles were selected and combined for 3D auto‐refinement, which resulted in an overall 4.16 Å resolution map. Post­processing, including soft masking, yielded a map with a resolution of 3.98 Å.

## CONFLICT OF INTEREST

Feng Wang is the employee of Biortus Biosciences Co. Ltd. The other authors declare no conflict of interest.

## AUTHOR CONTRIBUTIONS

Lin Tang, Xiao‐Xue Yan, and Wenqing Xu conceived, designed, and supervised the study and wrote the manuscript. Lina Jia, Xin Xu, and Yan‐Ping Liu carried out constructs and purified proteins. Lina Jia performed ELISA. Yan‐Ping Liu and Li‐Fei Tian performed binding studies. Lina Jia and Chao Xiong performed pseudotype virus neutralization assays. Chao Xiong measured the blocking effect of antibodies by flow cytometry. Wenqing Xu, Dong Zhou, and Feng Wang contributed to sample preparation. Yan‐Ping Liu, Lina Jia, Zheng Liu, Xiao‐Xue Yan, Wenqing Xu, and Lin Tang collected, processed, and interpretated cryo‐EM data. All authors read and approved the contents of the manuscript.

## ETHICS STATEMENT

This project was permitted by the Independent Ethics Committee of Sichuan University. This research conforms to all the laws and ethical guidelines that apply in the country.

## Supporting information

Supporting Information

## Data Availability

Cryo‐EM density maps of the MA1ScFv/MA2Fab/MA5Fab/RBD_V367F_ and MA1ScFv/MA2Fab/RBD complexes have been deposited at the Electron Microscopy Data Bank with accession codes EMD‐30861 and EMD‐30863, respectively.

## References

[1] Vankadari N , Wilce JA . Emerging COVID‐19 coronavirus: glycan shield and structure prediction of spike glycoprotein and its interaction with human CD26. Emerg Microbes Infect. 2020;9(1):601–604. 32178593 10.1080/22221751.2020.1739565 PMC7103712

[2] Chen L , Xiong J , Bao L , Shi Y . Convalescent plasma as a potential therapy for COVID‐19. Lancet Infect Dis. 2020;20(4):398–400. 32113510 10.1016/S1473-3099(20)30141-9 PMC7128218

[3] Cao XT . COVID‐19: immunopathology and its implications for therapy. Nat Rev Immunol. 2020;20(5):269–270. 32273594 10.1038/s41577-020-0308-3 PMC7143200

[4] Ju B , Zhang Q , Ge JW , et al. Human neutralizing antibodies elicited by SARS‐CoV‐2 infection. Nature. 2020;584(7819):115–119. 32454513 10.1038/s41586-020-2380-z

[5] Cao YL , Su B , Guo XH , et al. Potent neutralizing antibodies against SARS‐CoV‐2 identified by high‐throughput single‐cell sequencing of convalescent patients' B cells. Cell. 2020;182(1):73–84. 32425270 10.1016/j.cell.2020.05.025 PMC7231725

[6] Shi R , Shan C , Duan XM , et al. A human neutralizing antibody targets the receptor‐binding site of SARS‐CoV‐2. Nature. 2020;584(7819):120–124. 32454512 10.1038/s41586-020-2381-y

[7] Baum A , Fulton BO , Wloga E , et al. Antibody cocktail to SARS‐CoV‐2 spike protein prevents rapid mutational escape seen with individual antibodies. Science. 2020;369(6506):1014–1018. 32540904 10.1126/science.abd0831 PMC7299283

[8] Rogers TF , Zhao FZ , Huang DL , et al. Isolation of potent SARS‐CoV‐2 neutralizing antibodies and protection from disease in a small animal model. Science. 2020;369(6506):956–963. 32540903 10.1126/science.abc7520 PMC7299280

[9] Piccoli L , Park YJ , Tortorici MA , et al. Mapping neutralizing and immunodominant sites on the SARS‐CoV‐2 spike receptor‐binding domain by structure‐guided high‐resolution serology. Cell. 2020;183(4):1024–1042. 32991844 10.1016/j.cell.2020.09.037 PMC7494283

[10] Tortorici MA , Beltramello M , Lempp FA , et al. Ultrapotent human antibodies protect against SARS‐CoV‐2 challenge via multiple mechanisms. Science. 2020;370(6519):950–957. 32972994 10.1126/science.abe3354 PMC7857395

[11] Barnes CO , Jette CA , Abernathy ME , et al. SARS‐CoV‐2 neutralizing antibody structures inform therapeutic strategies. Nature. 2020;588(7839):682–687. 33045718 10.1038/s41586-020-2852-1 PMC8092461

[12] Yan RH , Zhang YY , Li YN , Xia L , Guo YY , Zhou Q . Structural basis for the recognition of SARS‐CoV‐2 by full‐length human ACE2. Science. 2020;367(6485):1444–1448. 32132184 10.1126/science.abb2762 PMC7164635

[13] Shang J , Ye G , Shi K , et al. Structural basis of receptor recognition by SARS‐CoV‐2. Nature. 2020;581(7807):221–224. 32225175 10.1038/s41586-020-2179-y PMC7328981

[14] Lan J , Ge JW , Yu JF , et al. Structure of the SARS‐CoV‐2 spike receptor‐binding domain bound to the ACE2 receptor. Nature. 2020;581(7807):215–220. 32225176 10.1038/s41586-020-2180-5

[15] Walls AC , Park YJ , Tortorici MA , Wall A , McGuire AT , Veesler D . Structure, function, and antigenicity of the SARS‐CoV‐2 spike glycoprotein. Cell. 2020;181(1):281–292. 32155444 10.1016/j.cell.2020.02.058 PMC7102599

[16] Song WF , Gui M , Wang XQ , et al. Cryo‐EM structure of the SARS coronavirus spike glycoprotein in complex with its host cell receptor ACE2. PLoS Pathog. 2018;14(8):e1007236. 30102747 10.1371/journal.ppat.1007236 PMC6107290

[17] Hoffmann M , Kleine‐Weber H , Schroeder S , et al. SARS‐CoV‐2 cell entry depends on ACE2 and TMPRSS2 and is blocked by a clinically proven protease inhibitor. Cell. 2020;181(2):271–280. 32142651 10.1016/j.cell.2020.02.052 PMC7102627

[18] He YX , Lu H , Siddiqui P , Zhou YS , Jiang SB . Receptor‐binding domain of severe acute respiratory syndrome coronavirus spike protein contains multiple conformation‐dependent epitopes that induce highly potent neutralizing antibodies. J Immunol. 2005;174(8):4908–4915. 15814718 10.4049/jimmunol.174.8.4908

[19] Yuan M , Wu NC , Zhu XY , et al. A highly conserved cryptic epitope in the receptor binding domains of SARS‐CoV‐2 and SARS‐CoV. Science. 2020;368(6491):630–633. 32245784 10.1126/science.abb7269 PMC7164391

[20] Prabakaran P , Gan JH , Feng Y , et al. Structure of severe acute respiratory syndrome coronavirus receptor‐binding domain complexed with neutralizing antibody. J Biol Chem. 2006;281(23):15829–15836. 16597622 10.1074/jbc.M600697200 PMC8099238

[21] Kubo H , Yamada YK , Taguchi F . Localization of neutralizing epitopes and the receptor‐binding site within the amino‐terminal 330 amino acids of the murine coronavirus spike protein. J Virol. 1994;68(9):5403–5410. 7520090 10.1128/jvi.68.9.5403-5410.1994 PMC236940

[22] Wang LS , Shi W , Chappell JD , et al. Importance of neutralizing monoclonal antibodies targeting multiple antigenic sites on the middle east respiratory syndrome coronavirus spike glycoprotein to avoid neutralization escape. J Virol. 2018;92(10):e02002–e02017. 29514901 10.1128/JVI.02002-17 PMC5923077

[23] Tian XL , Li C , Huang AL , et al. Potent binding of 2019 novel coronavirus spike protein by a SARS coronavirus‐specific human monoclonal antibody. Emerg Microbes Infect. 2020;9(1):382–385. 32065055 10.1080/22221751.2020.1729069 PMC7048180

[24] Smith TRF , Patel A , Ramos S , et al. Immunogenicity of a DNA vaccine candidate for COVID‐19. Nat Commun. 2020;11(1):2601. 32433465 10.1038/s41467-020-16505-0 PMC7239918

[25] Zhu FC , Li YH , Guan XH , et al. Safety, tolerability, and immunogenicity of a recombinant adenovirus type‐5 vectored COVID‐19 vaccine: a dose‐escalation, open‐label, non‐randomised, first‐in‐human trial. Lancet. 2020;395(10240):1845–1854. 32450106 10.1016/S0140-6736(20)31208-3 PMC7255193

