# Supplementary material for: Potent neutralizing RBD‐specific antibody cocktail against SARS‐CoV‐2 and its mutant
Source: MedComm (2020). 2021 Jun 24;2(3):442–52. doi: 10.1002/mco2.79 (PMC8441738; doi:10.1002/mco2.79)
Supplement: Supplementary file 1 — Supporting Information [file MCO2-2-442-s001.pdf]

Potent neutralizing RBD-specific antibody cocktail against SARS-CoV-2 and its mutant

Lina Jia [1,#] | Yan-Ping Liu [2,#] | Li-Fei Tian [2] | Chao Xiong [1] | Xin Xu [1] | Honge Qu [1] | Weixi Xiong [1] | Dong Zhou [1] | Feng Wang [4] | Zheng Liu [3] | Xiao-Xue Yan [2,*] | Wenqing Xu [2, 5,*] | Lin Tang [1,*]

[1] Department of Neurology, State Key Lab of Biotherapy and Cancer center, West China Hospital, Sichuan University and Collaborative Innovation Center for Biotherapy, Chengdu, 610041 Sichuan, China;

[2] National Laboratory of Biomacromolecules, Chinese Academy of Sciences (CAS) Center for Excellence in Biomacromolecules, Institute of Biophysics, Chinese Academy of Sciences, 100101 Beijing, China;

[3] School of life and health, Kobilka Institute of Innovative Drug Discovery, the Chinese University of Hong Kong (Shenzhen), China;

[4] Wuxi Biortus Biosciences Co. Ltd., 6 Dongsheng West Road, Jiangyin, 214437, China;

[5] Shanghai Institute for Advanced Immunochemical Studies and School of Life Science and Technology, ShanghaiTech University, Shanghai, China.

*Correspondence: Xiao-Xue Yan (snow@ibp.ac.cn) or Wenqing Xu (xuwq2@shanghaitech.edu.cn) or Lin Tang (ltang@scu.edu.cn) .

[#]These authors contributed equally: Lina Jia, Yan-Ping Liu.

Keywords: SARS-CoV-2, receptor-binding domain, neutralizing antibody, cocktail, epitopes group, Cyro-EM

SUPPORTING MATERIALS

1 | MATERIALS AND METHODS

1.1 | In vitro expression of mAbs

The genes encoding the heavy and light chains of the identified antibodies were separately cloned into expression vectors containing IgG1 constant regions, and the vectors were transiently transfected into 293F cells using polyethylenimine (Sigma). After 72 h, the antibodies secreted into the supernatant were collected and captured by protein A Sepharose (GE Healthcare). The bound antibodies were eluted and further purified by gel filtration chromatography using a Superdex 200 high-performance column (GE Healthcare). The purified antibodies were either used in binding or neutralizing assays.

1.2 | ELISA analysis

ELISA plates were coated with SARS-CoV-2 RBD and S protein at 0.5 μg/mL in phosphate-buffered saline at 4°C overnight. After standard washing and blocking, 100 μL antibody dilution or supernatant of hybridoma clone was added to each well. After 2 h incubation at room temperature, the plates were washed and incubated with 0.08 μg/mL goat anti-human IgG (H + L)/horseradish peroxidase (HRP) for 1 h at room temperature. The reaction was visualized by adding the substrate 3,3′,5,5′-tetramethylbenzidine (TMB). Absorbance at 450 nm was measured using an ELISA plate reader.

An ELISA plate was coated with recombinant RBD-His at 1 μg/mL and then treated for 1 h at 37°C with 10 mM DTT followed by washing to determine the effect of disulfide bond reduction on the binding of RBD-specific mAbs. Then, the wells were treated with 50 mM iodoacetamide for 1 h at 37°C. After washing, standard ELISA was performed as described above.

Competitive ELISA was performed to determine the inhibitory activity of RBD-specific mAbs on the binding of biotinylated mAbs to RBD. Briefly, the wells of ELISA plates were coated with the RBD-His tag at 1 μg/mL. A mixture containing 50 μg/mL unlabeled mAb and

1 μg/mL biotinylated mAb was added, followed by incubation at 37°C for 1 h. The binding of biotinylated mAbs was detected after adding HRP-conjugated streptavidin (Zymed Laboratories) and TMB sequentially.

1.3 | Epitope binding by SPR

For epitope mapping, two different antibodies were sequentially injected and monitored for binding activity using SPR (Biacore T100; GE Healthcare). The sensorgrams showed distinct binding patterns when pairs of testing antibodies were sequentially applied to the purified SARS-CoV-2 RBD or SARS-CoV-2 RBD_{V367F} covalently immobilized onto a CM5 sensor chip. SARS-CoV-2 RBD or SARS-CoV-2 RBD_{V367F} was immobilized to a CM5 sensor chip via amine group for a final RU of approximately 1500. The first antibodies were injected onto the chip until a binding steady state was reached, and the second antibodies were then injected for 180 s. The level of reduction in RU comparing with or without prior antibody incubation is the key criterion to determine that the two mAbs recognize separate or closely situated epitopes.

1.4 | Pseudovirus neutralization assays

SARS-CoV-2 and its mutant SARS-CoV-2(V367F) pseudovirus were generated by co-transfection of HIV backbones expressing firefly luciferase (pNL43R-E-luciferase) and pcDNA3.1 (Invitrogen) expression vectors encoding the respective S and S_{V367F} proteins into 293T cells (American Type Culture Collection). Viral supernatants were collected 48 h later. Viral titers were measured as luciferase activity in relative light units (Bright-Glo Luciferase Assay Vector System; Promega Biosciences). Neutralization assays were performed by incubating pseudoviruses with serial dilutions of purified mAbs or mAb cocktails at 37°C for 1 h. Cells for SARS-CoV-2 and SARS-CoV-2 pseudovirus (~15,000 per well) were added in duplicate to the virus–antibody mixture. The IC_{50} of the evaluated mAbs was determined by luciferase activity 48 h after exposure to the virus–antibody mixture using GraphPad Prism 7 (GraphPad Software).

1.5 | Live SARS-CoV-2 neutralization assay

A neutralization assay of live SARS-CoV-2 was performed using the cytopathic effect (CPE) assay. Briefly, threefold serial dilutions of mAbs were mixed with a volume of 100 PFU authentic SARS-CoV-2 and incubated at 37°C for 1 h. The mixture was added to a monolayer of Vero-E6 cells in a 96-well plate and incubated for 1 h at 37°C. The supernatant was removed, and 200 μL Dulbecco's modified Eagle's medium (supplied with 3% fetal bovine serum) was added to the infected cells. After incubation at 37°C supplied with 5% CO_2 for 3 days, all wells were examined for the CPE effect. All experiments were performed in a Biosafety Level 3 facility.

1.6 | Cryo-EM sample preparation and data collection

Three microliters of the MA1ScFv/MA2Fab/MA5Fab/RBD_{V367F} complex (0.6 mg/mL) were loaded onto a freshly glow discharge (50 s at 20 mA) 1.2/1.3 UltrAuFoil (300 mesh) before plunge freezing using a vitrobot MarKIV (Thermo Fisher Scientific) with a blot force of 0 and 6 s blot time at 100% humidity at 22°C.

Data were acquired on an FEI Titan Krios transmission electron microscope operated at 300 kV and equipped with a Gatan K2 Summit direct detector and Gatan Quantum GIF energy filter. Movies were collected using SerialEM automated data collection software at a magnification of × 22,500 with a pixel size of 1.02 Å. The dose rate was adjusted to 8 counts/pixel/s, and each movie was fractioned in 40 frames of 200 ms. Three thousand micrographs were collected with a defocus range between −0.5 and −3.0 μm.

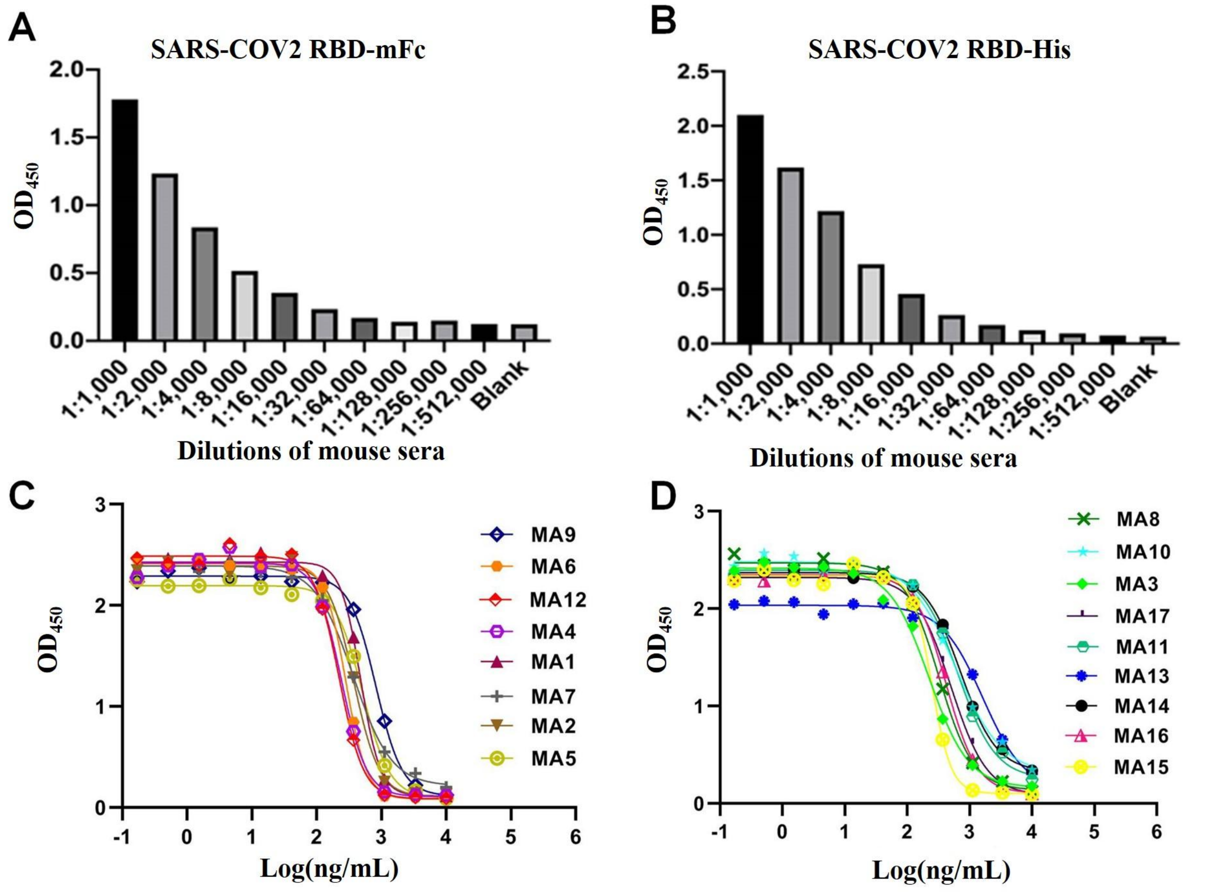

Fig. (S1). Serological responses to the SARS-CoV-2 RBD and identification of 17 RBD-specific mAbs that inhibit binding of the RBD to ACE2. (A,B) Serological antibody responses to the RBD-mFc and RBD-His. The plasma sample from mice six times after vaccination with RBD-mFc was evaluated for binding to the RBD coated on the ELISA plates. (C,D) Inhibitory curves of each mAb competing with ACE2 binding.

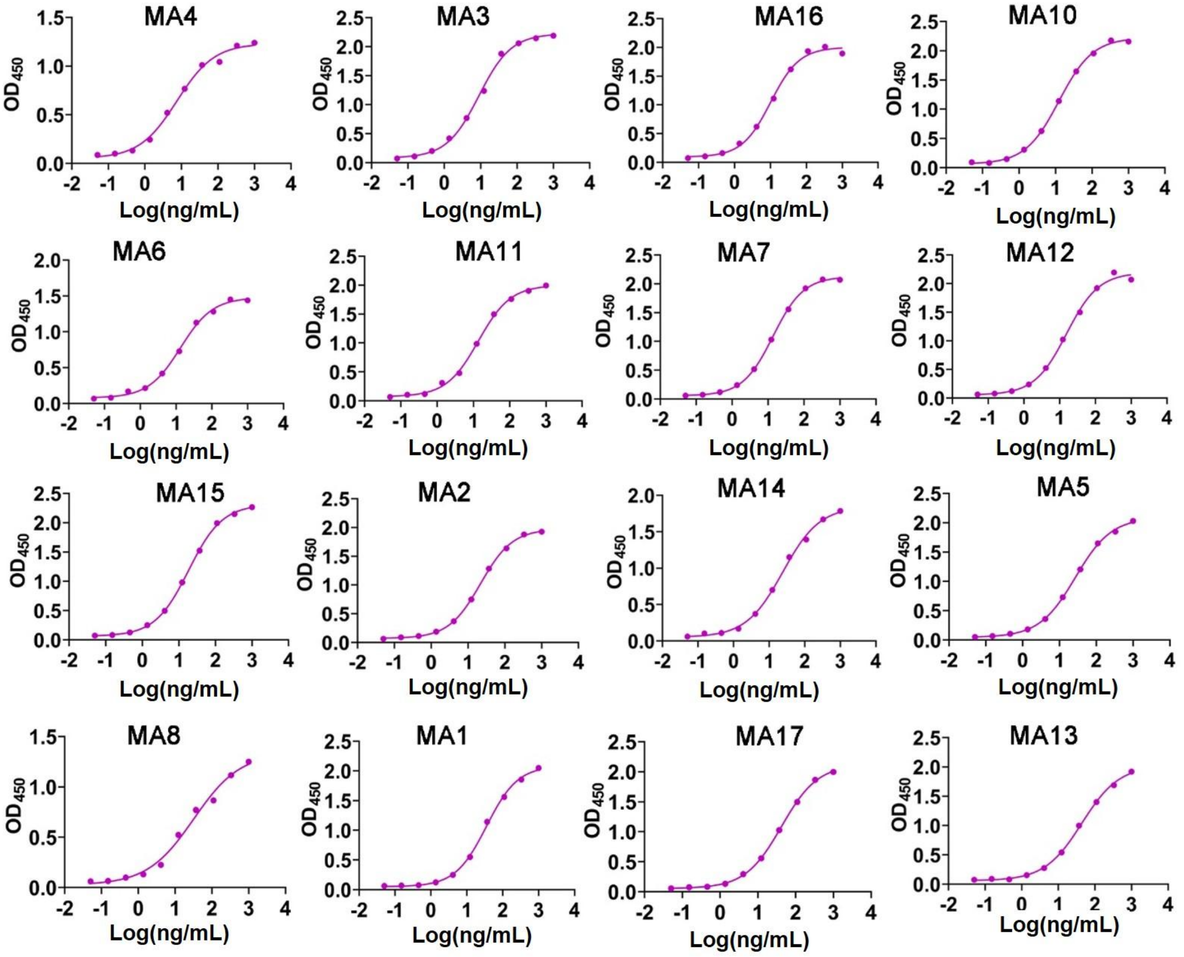

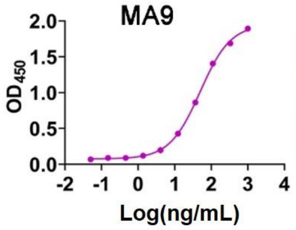

mAbs	MA4	MA3	MA16	MA11	MA10	MA6	MA7	MA12	MA15
EC50 (nM)	0.052	0.049	0.074	0.086	0.092	0.096	0.095	0.107	0.127
mAbs	MA2	MA14	MA5	MA8	MA1	MA17	MA13	MA9	
EC50 (nM)	0.151	0.168	0.188	0.221	0.234	0.290	0.304	0.347	

Fig. (S2). Binding activities of RBD-specific mAbs to SARS-CoV-2 RBD by ELISA. Purified mAbs are applied to ELISA plates coated with trimeric RBD to measure the binding activity.

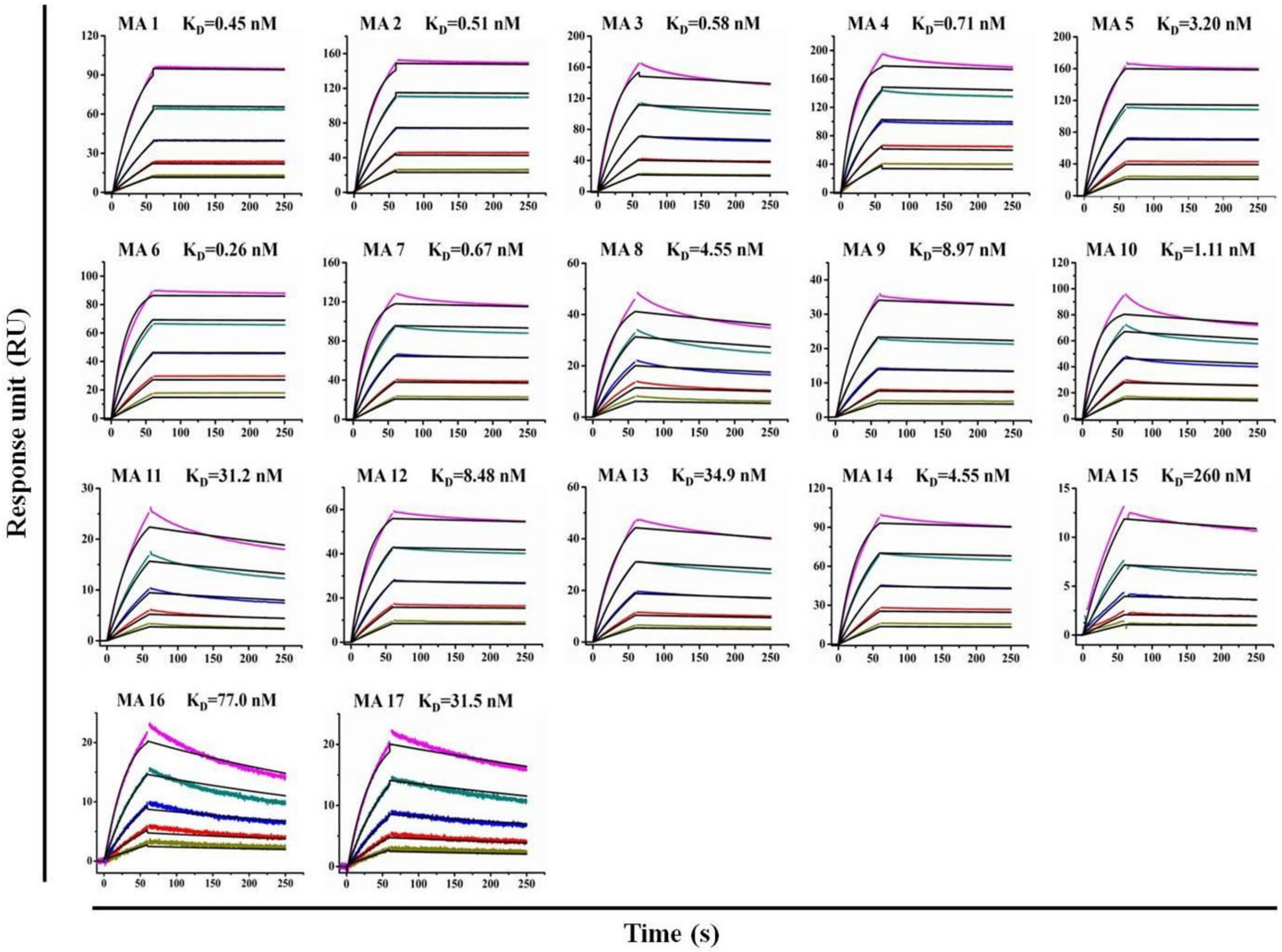

Fig. (S3). Binding kinetics of RBD-specific mAbs with SARS-CoV-2 RBD. The purified soluble SARS-CoV-2 RBD was covalently immobilized onto a CM5 sensor chip followed by injection of each antibody with five different concentrations. The black line indicates the experimentally derived curves, and the red lines represent the fitted curves based on the experimental data.

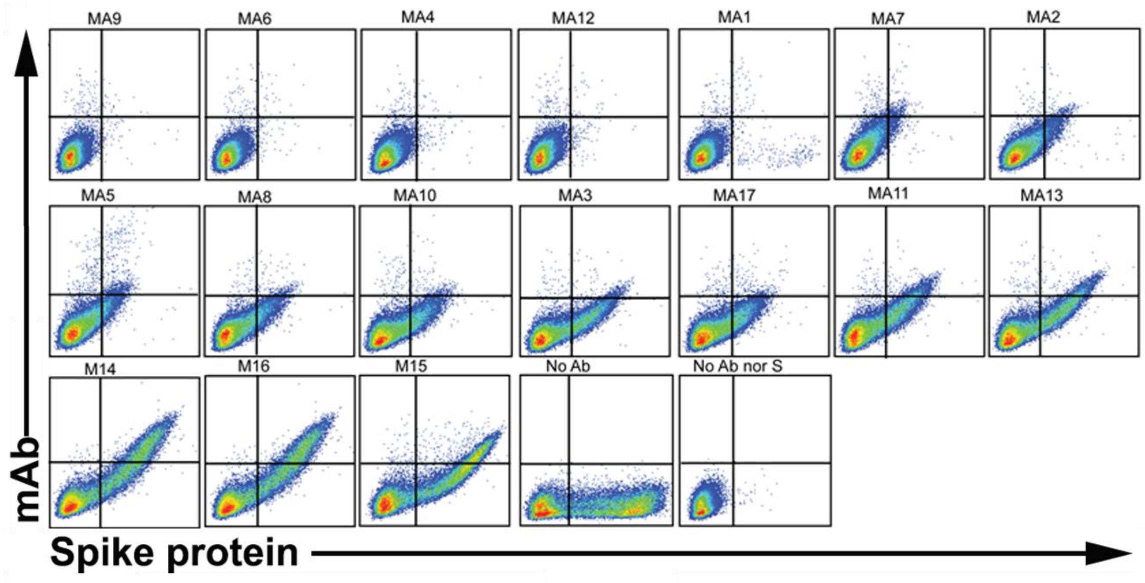

Fig. (S4). Inhibition of S protein binding to human ACE2 overexpressing on 293T cells determined by flow cytometry. After preincubation of S protein with the indicated mAb at a final concentration of 10 µg/mL, the mAb-S mixture was added to the ACE2-expressing cells. Cells were stained with anti-human IgG FITC (mAb binding; *x*-axis) and anti-His (S binding; *y*-axis). One representative of three experiments is shown.

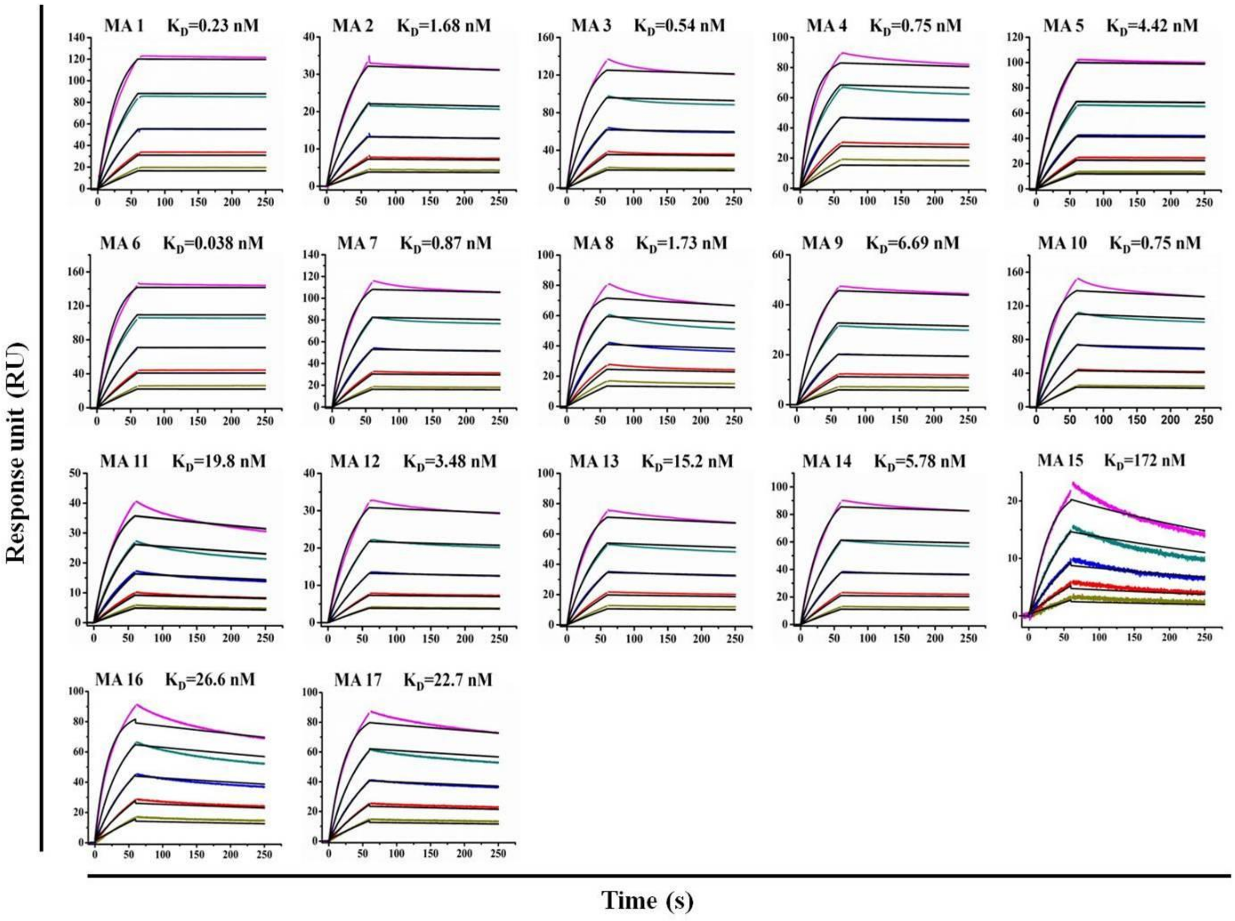

Fig. (S5). Binding kinetics of RBD-specific mAbs with SARS-CoV-2 RBD_{V367F}. The purified soluble SARS-CoV-2 RBD_{V367F} was covalently immobilized onto a CM5 sensor chip followed by injection of each antibody with five different concentrations. The black line indicates the experimentally derived curves, and the colored lines represent the fitted curves based on the experimental data.

Group	mAbs	MA1	MA9	MA4	MA3	MA2	MA8	MA7	MA10	MA11	MA14	MA13	MA6	MA12	MA15	MA5	MA16	MA17
I	MA1	77.5	74.8	33.1	10.0	23.7	31.3	15.8	22.9	32.2	44.0	68.4	27.3	50.4	45.3	7.8	-6.1	15.6
II	MA9	62.9	76.0	56.9	75.2	85.7	85.7	83.0	93.6	87.7	93.5	75.9	31.8	62.9	64.2	12.3	9.2	14.8
II	MA4	32.5	92.4	85.9	95.3	93.4	95.0	95.1	95.7	89.6	95.7	95.6	71.0	55.6	49.7	19.8	15.0	13.9
II	MA3	13.8	82.8	56.8	87.9	84.0	91.8	91.1	92.0	94.1	94.4	84.9	38.0	44.6	42.9	3.7	-14.0	32.3
II	MA2	19.8	82.9	76.3	92.1	89.4	94.1	94.2	93.2	94.3	95.9	91.8	46.7	60.7	64.5	-5.7	-1.3	16.0
II	MA8	8.7	78.7	59.6	88.4	76.0	89.3	87.5	92.7	92.9	94.6	86.1	25.6	41.4	40.5	4.3	0.6	4.3
II	MA7	4.7	70.5	44.7	83.6	69.0	85.7	87.2	95.2	91.2	94.2	76.5	21.5	40.8	40.2	-4.3	2.3	-8.6
II	MA10	13.9	69.0	53.1	85.9	78.4	90.0	88.6	95.0	92.8	95.0	68.0	32.3	44.3	42.5	11.7	-8.0	22.4
II	MA11	6.4	62.4	27.9	62.5	59.5	75.5	70.4	93.5	84.0	90.4	54.4	16.8	37.0	34.0	12.6	6.0	19.9
II	MA14	18.3	67.9	41.8	78.3	63.1	81.4	74.5	94.1	88.4	92.7	62.1	20.3	37.6	35.7	19.5	11.3	21.6
II	MA13	7.0	28.9	11.9	36.5	33.2	50.0	47.1	86.5	63.0	94.2	72.5	15.0	23.1	24.5	11.0	-1.5	21.6
III	MA6	89.9	91.7	90.2	93.9	93.1	95.8	95.5	96.3	96.4	94.9	95.3	88.4	90.1	92.9	86.0	90.4	26.3
III	MA12	65.1	71.3	39.7	72.8	74.0	81.4	82.4	93.8	89.4	93.2	79.0	28.4	76.6	77.3	60.6	74.6	61.4
III	MA15	65.8	89.9	52.0	77.7	83.1	87.9	89.7	95.3	92.8	94.1	89.1	60.4	89.6	89.6	65.0	89.3	66.7
IV	MA5	17.0	25.8	30.6	26.2	13.2	29.7	41.6	49.5	41.0	55.8	24.1	39.0	82.9	76.8	61.5	76.4	70.3
IV	MA16	8.8	38.8	40.2	26.3	39.1	31.4	36.5	27.6	38.9	63.9	21.7	19.7	52.5	50.7	42.0	59.1	55.9
IV	MA17	11.5	15.3	31.2	21.9	16.3	25.8	21.3	43.8	20.8	41.2	15.8	10.6	51.4	52.1	52.4	53.0	78.7

Fig. (S6). Epitope mapping through competitive binding measured by ELISA and SPR. His-tagged SARS-CoV-2 RBD was coated onto ELISA plates, and the unlabeled antibody (column) was applied in the presence of each biotinylated antibody (row). The percent reduction of biotinylated antibody binding to RBD was measured as the inhibitory rates shown in this Table.

A

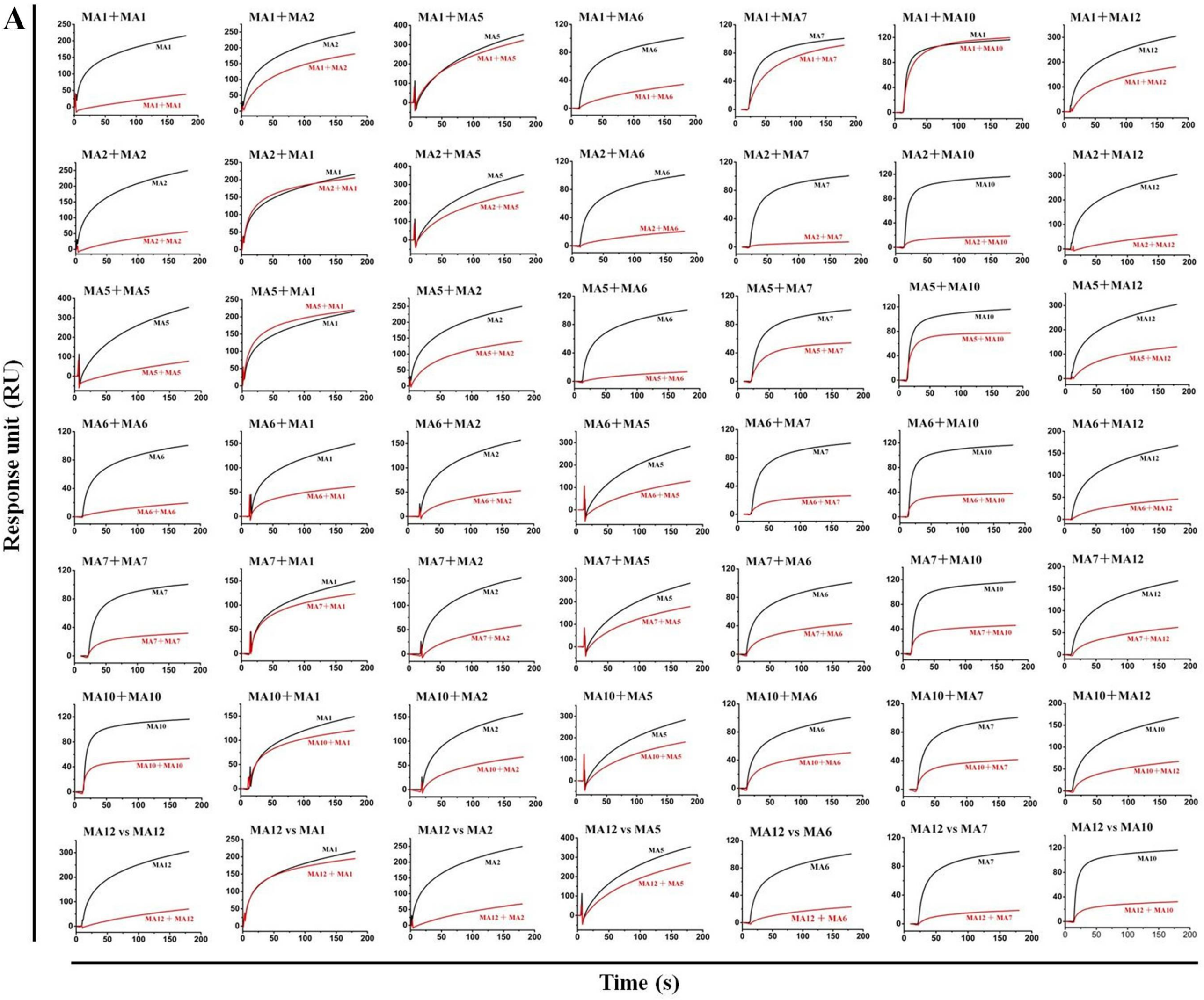

B

mAbs	MA1	MA2	MA5	MA6	MA7	MA10	MA12
MA1		−	−	++	−	−	+
MA2	−		−	+++	+++	+++	+++
MA5	−	+		+++	+	+	+
MA6	++	+++	++		++	++	++
MA7	−	++	+	++		++	++
MA10	−	++	+	++	++		++
MA12	−	++	−	++	+++	++	

Fig. (S7). mAb epitope mapping through competitive binding measured by SPR. (A) The sensorgrams showed distinct binding patterns when pairs of testing antibodies were

sequentially applied to the purified SARS-CoV-2 RBD covalently immobilized onto a CM5 sensor chip. The level of reduction in RU comparing with or without prior mAb incubation is the key criterion to determine two mAbs that recognize separate or closely situated epitopes. Results are representative of two independent experiments. (B) For epitope mapping, the summary of the mentioned competition in Extended Data Figure 4 is as follows: "+++," >80% competition among same epitopes; "++," 50%–80% overlapping epitopes; "+," 25% to 50% overlapping epitopes; and "−," <25% distinct epitopes.

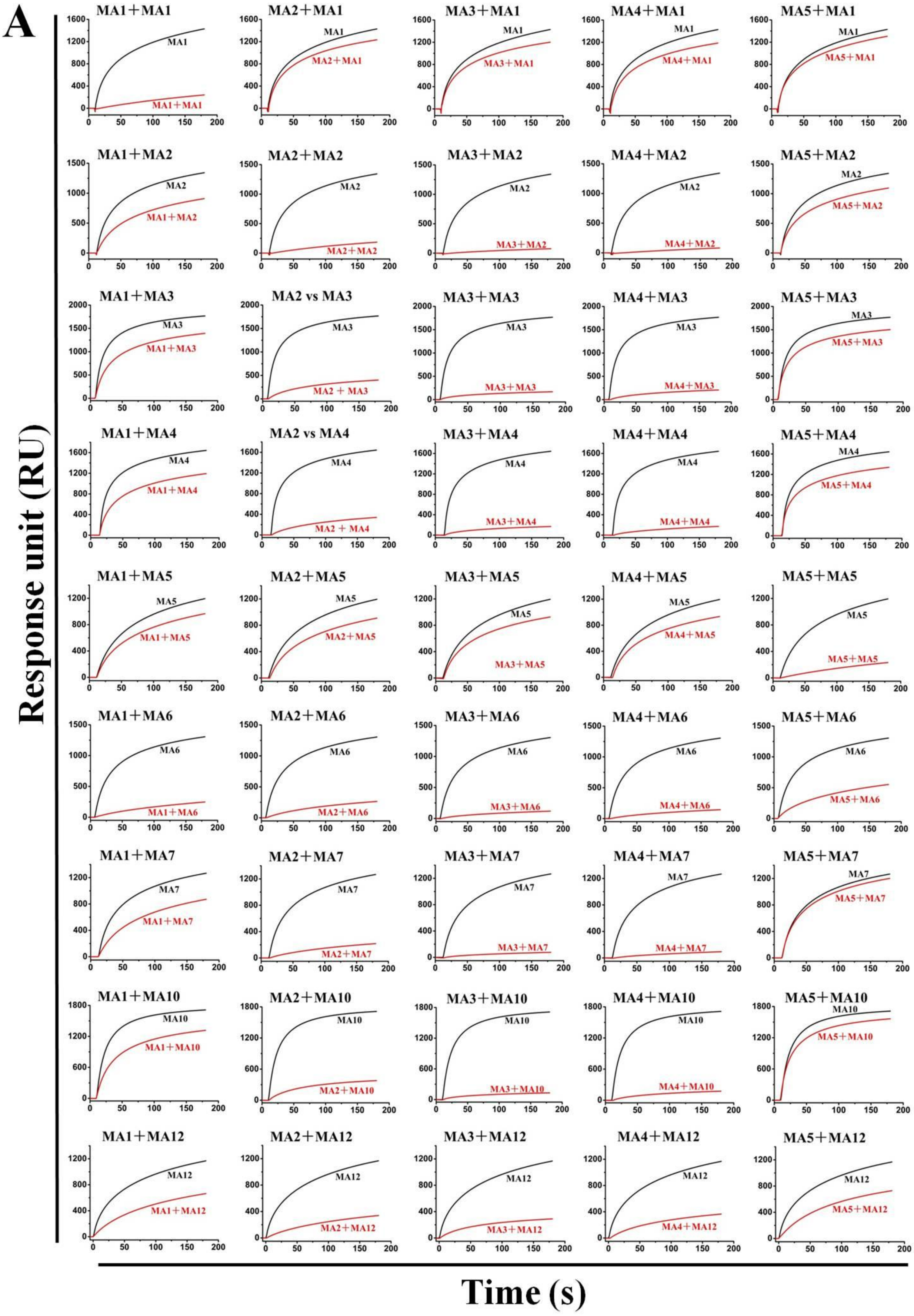
A
MA1+MA1
MA2+MA1
MA3+MA1
MA4+MA1
MA5+MA1
MA1+MA2
MA2+MA2
MA3+MA2
MA4+MA2
MA5+MA2
MA1+MA3
MA2 vs MA3
MA3+MA3
MA4+MA3
MA5+MA3
MA1+MA4
MA2 vs MA4
MA3+MA4
MA4+MA4
MA5+MA4
MA1+MA5
MA2+MA5
MA3+MA5
MA4+MA5
MA5+MA5
MA1+MA6
MA2+MA6
MA3+MA6
MA4+MA6
MA5+MA6
MA1+MA7
MA2+MA7
MA3+MA7
MA4+MA7
MA5+MA7
MA1+MA10
MA2+MA10
MA3+MA10
MA4+MA10
MA5+MA10
MA1+MA12
MA2+MA12
MA3+MA12
MA4+MA12
MA5+MA12
Response unit (RU)
Time (s)

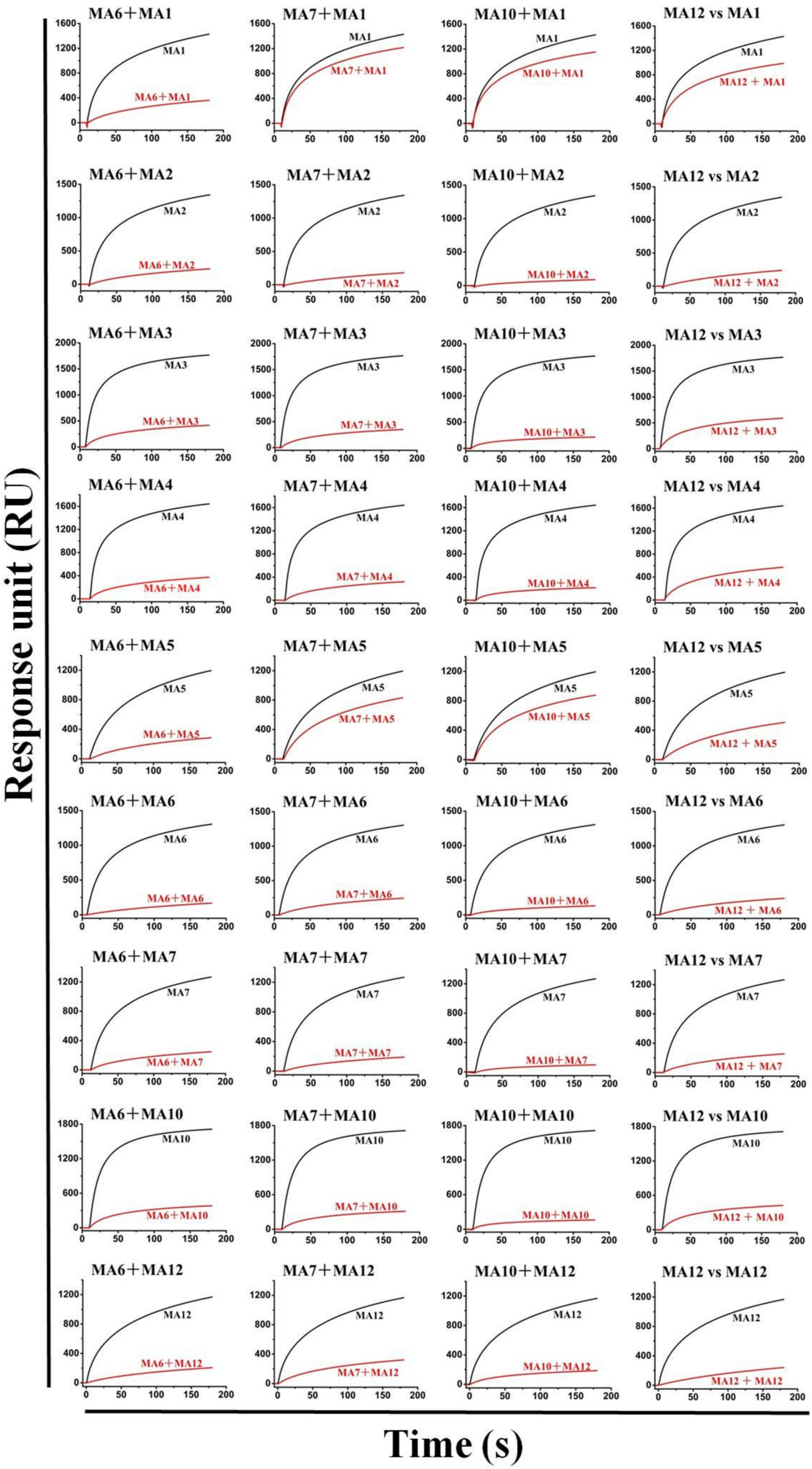

Response unit (RU)
Time (s)
MA6+MA1
MA1
MA6+MA1
MA7+MA1
MA1
MA7+MA1
MA10+MA1
MA1
MA10+MA1
MA12 vs MA1
MA1
MA12 + MA1
MA6+MA2
MA2
MA6+MA2
MA7+MA2
MA2
MA7+MA2
MA10+MA2
MA2
MA10+MA2
MA12 vs MA2
MA2
MA12 + MA2
MA6+MA3
MA3
MA6+MA3
MA7+MA3
MA3
MA7+MA3
MA10+MA3
MA3
MA10+MA3
MA12 vs MA3
MA3
MA12 + MA3
MA6+MA4
MA4
MA6+MA4
MA7+MA4
MA4
MA7+MA4
MA10+MA4
MA4
MA10+MA4
MA12 vs MA4
MA4
MA12 + MA4
MA6+MA5
MA5
MA6+MA5
MA7+MA5
MA5
MA7+MA5
MA10+MA5
MA5
MA10+MA5
MA12 vs MA5
MA5
MA12 + MA5
MA6+MA6
MA6
MA6+MA6
MA7+MA6
MA6
MA7+MA6
MA10+MA6
MA6
MA10+MA6
MA12 vs MA6
MA6
MA12 + MA6
MA6+MA7
MA7
MA6+MA7
MA7+MA7
MA7
MA7+MA7
MA10+MA7
MA7
MA10+MA7
MA12 vs MA7
MA7
MA12 + MA7
MA6+MA10
MA10
MA6+MA10
MA7+MA10
MA10
MA7+MA10
MA10+MA10
MA10
MA10+MA10
MA12 vs MA10
MA10
MA12 + MA10
MA6+MA12
MA12
MA6+MA12
MA7+MA12
MA12
MA7+MA12
MA10+MA12
MA12
MA10+MA12
MA12 vs MA12
MA12
MA12 + MA12

B

mAbs	MA1	MA2	MA3	MA4	MA5	MA6	MA7	MA10	MA12
**MA1**		−	−	−	−	+++	−	−	+
**MA2**	−		+++	+++	−	+++	+++	++	++
**MA3**	−	+++		+++	−	+++	+++	+++	++
**MA4**	−	+++	+++		−	+++	+++	+++	++
**MA5**	−	−	−	−		++	−	−	+
**MA6**	++	+++	+++	+++	++		+++	++	+++
**MA7**	−	+++	+++	+++	−	+++		+++	++
**MA10**	−	+++	+++	+++	−	+++	+++		+++
**MA12**	−	+++	++	++	+	+++	+++	++	

Figure. (S8). Epitope mapping through competitive binding measured by SPR. MA1, MA2, and MA5 bind simultaneously to SARS-CoV-2 RBD$_{V367F}$. (A) The sensorgrams showed distinct binding patterns when pairs of testing antibodies were sequentially applied to the purified RBD$_{V367F}$, which was covalently immobilized onto a CM5 sensor chip. The level of reduction in RU comparing with or without prior antibody incubation is the key criterion to determine two mAbs that recognize separate or closely situated epitopes. (B) Summary of the abovementioned competition: "+++," >80% competition; "++," 50% to 80%; "+," 25% to 50%; and "–," <25%. Results in (a) are representatives of two independent experiments.

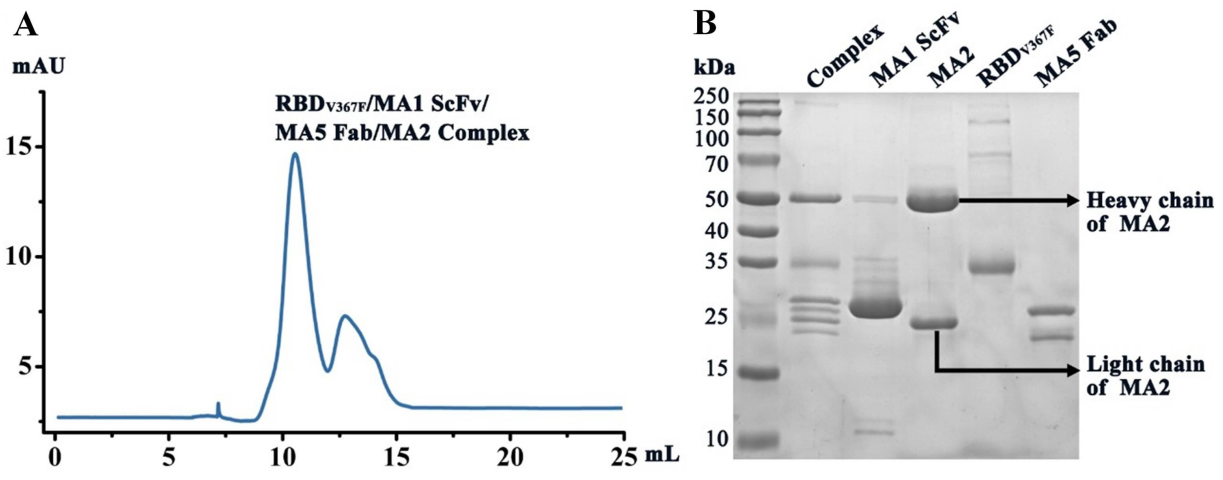

Fig. (S9). MA1, MA2 and MA5 bound to RBD_{V367F} simultaneously as revealed by the purified RBD_{V367F}/MA1scFv/MA2/MA5 Fab complex. The scFv of MA1, Fab of MA5, and full length of MA2 were incubated with recombinant RBD_{V367F} for 30 min before running through Superdex 200 column (A), and the peak fractions were concentrated and run on SDS-PAGE (B).

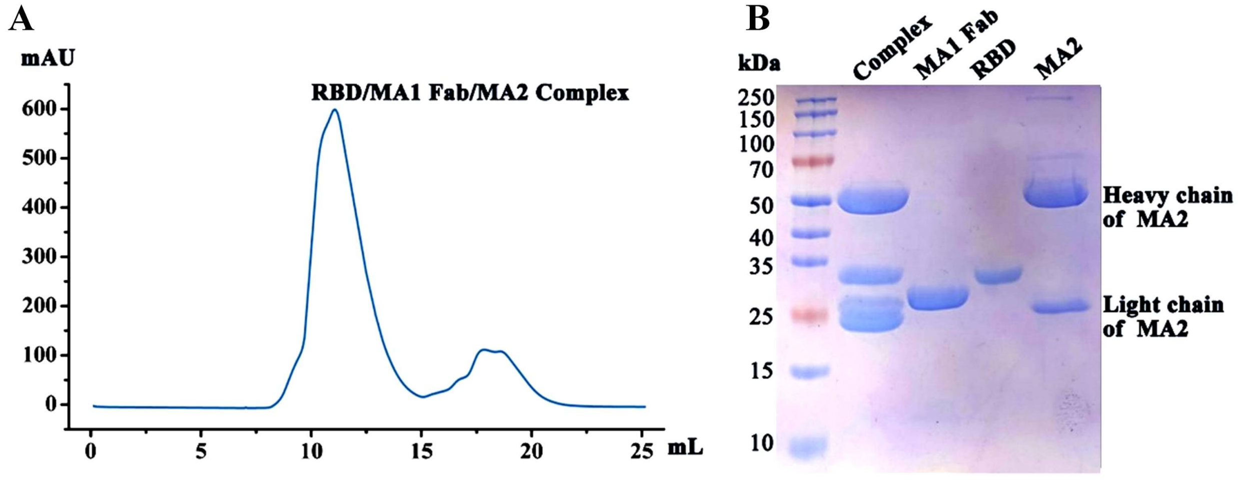

Fig. (S10). MA1 and MA2 bound to the RBD simultaneously as revealed by the purified RBD/MA1 Fab/MA2 complexes. The Fab of MA1 and full-length MA2 were incubated with recombinant RBD for 30 min before running through Superdex 200 column. The peak fractions were (A) concentrated and (B) run on SDS-PAGE.

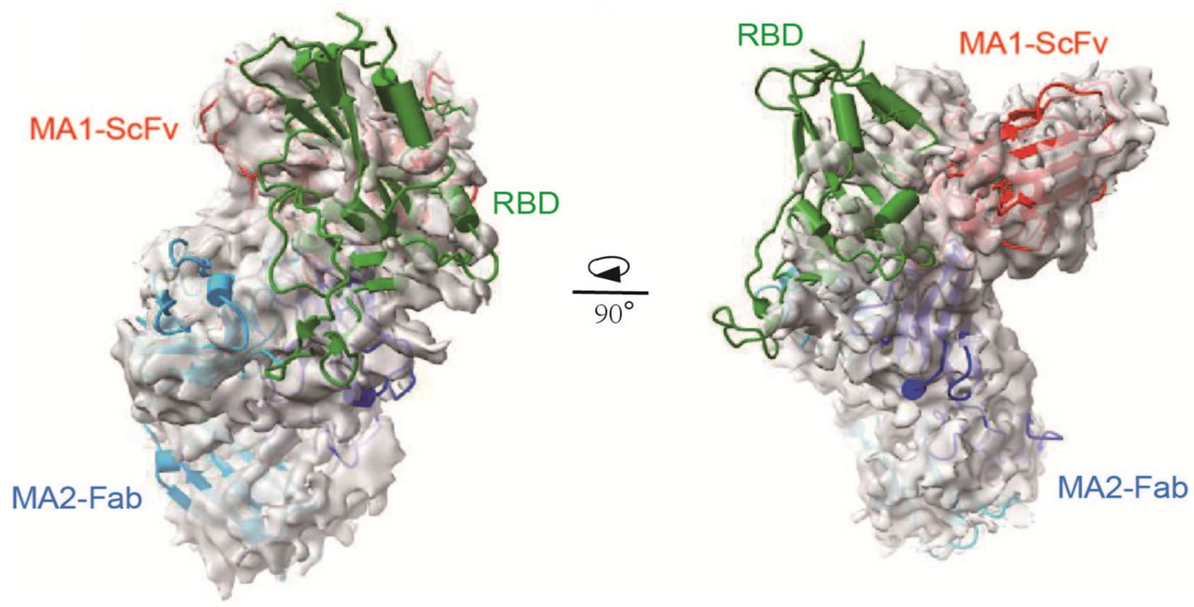

Fig. (S11). Cryo-EM structures of MA1ScFv/MA2Fab/RBD complex.

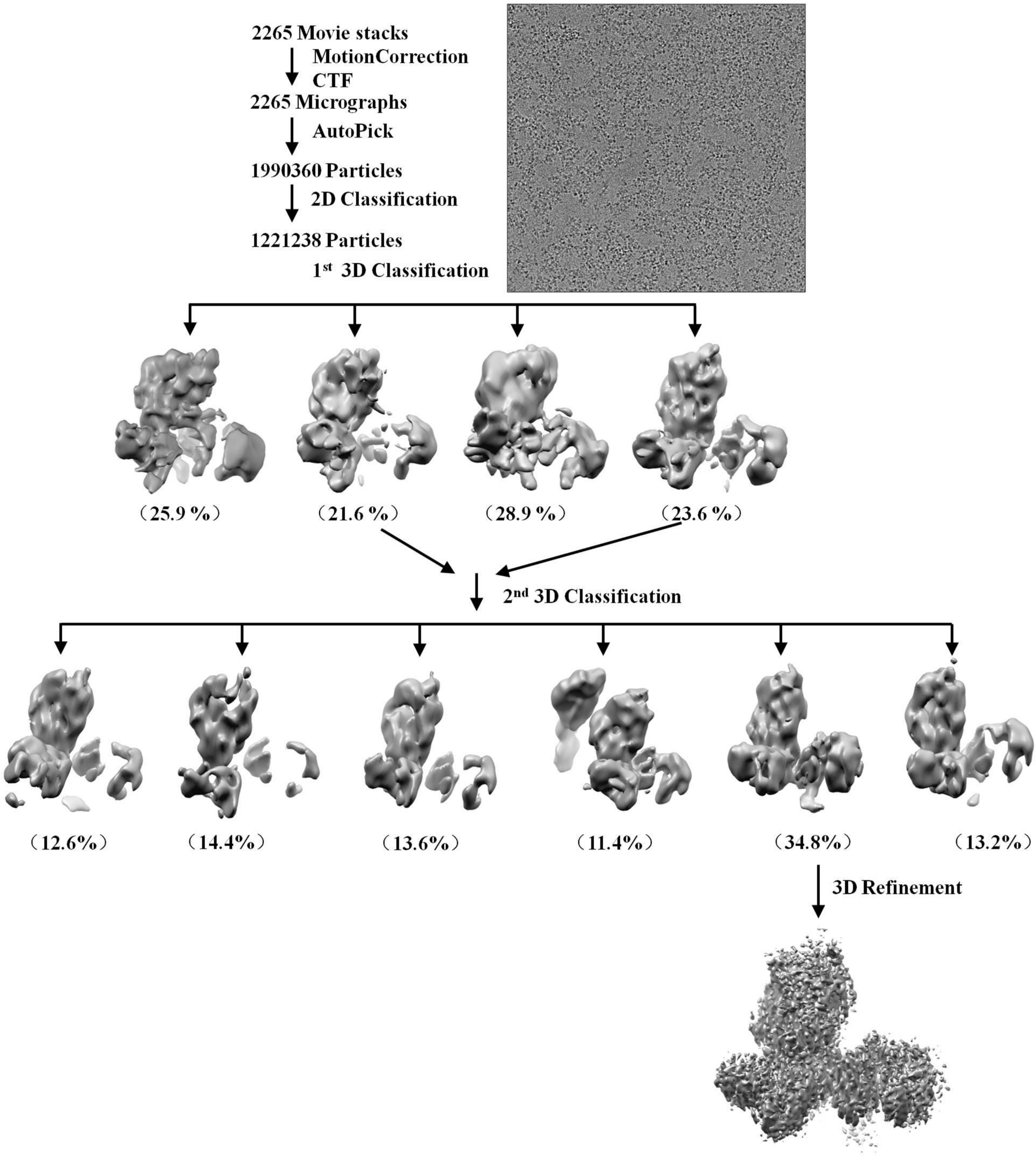

Fig. (S12). Flow chart for Cryo-EM data processing of MA1ScFv/MA2Fab/MA5Fab-/RBD$_{V367F}$ complex.

Table. S1. ELISA screening of high-potency ACE2 competition hybridoma clones.

The first screening identified the hybridoma clones binding to RBD with high affinity, and 74 clones were selected. Competitive ELISA was used to determine the clones that reduced the binding of S to the ACE2 receptor by more than 50% (highlighted).

	Clone	OD450 (S-ECD)	OD450 (ACE2-hFc)	Inhibition ratio		Clone	OD450 (S-ECD)	OD450 (ACE2-hFc)	Inhibition ratio
1	1B2	1.450	2.460	-38.67%	38	14F8	0.993	2.826	-59.30%
2	1B7	1.326	0.933	47.41%	39	15B5	1.113	3.21	-80.95%
3	1E9	1.296	1.516	14.54%	40	19F9	1.219	1.698	4.28%
4	1G10	1.398	2.362	-33.15%	41	20A3	1.469	2.201	-24.07%
5	2C11	1.336	0.834	52.99%	42	20E2	1.199	1.847	-4.11%
6	4H10	1.482	2.302	-29.76%	43	20H8	1.351	0.306	82.75%
7	5B5	1.482	0.17	90.42%	44	21A12	1.127	1.808	-1.92%
8	5B6	1.343	0.143	91.94%	45	23C9	0.997	0.426	75.99%
9	5F11	1.407	0.852	51.97%	46	24E2	1.063	2.436	-37.32%
10	5F12	1.437	2.454	-38.33%	47	24H10	1.160	1.867	-5.24%
11	6F4	1.420	1.762	0.68%	48	24H11	1.192	0.361	79.65%
12	6G3	1.491	2.42	-36.41%	49	25E4	0.916	1.761	0.73%
13	7C12	1.348	1.597	9.98%	50	26H8	0.960	0.533	69.95%
14	7D3	1.331	2.882	-62.46%	51	27A2	0.941	2.788	-57.16%
15	7E3	1.477	1.602	9.70%	52	29C1	0.993	1.221	31.17%
16	7F1	1.451	0.504	71.59%	53	29E2	0.932	1.489	16.07%
17	7F8	1.257	2.928	-65.05%	54	29H7	1.032	1.234	30.44%
18	8A2	1.371	1.166	34.27%	55	30A7	0.912	2.822	-59.08%
19	8B6	1.306	0.872	48.85%	56	30F6	1.024	1.225	30.95%
20	8D8	1.360	1.823	-2.76%	57	32F1	1.079	1.158	34.72%
21	9A1	1.572	1.54	13.19%	58	34A12	1.069	2.314	-30.44%
22	9A4	1.316	1.923	-8.40%	59	34D1	1.536	1.555	12.34%
23	9A10	1.347	1.303	26.55%	60	34G1	1.267	2.741	2.741
24	9B4	1.324	2.806	-58.17%	61	35B4	1.257	1.337	24.63%
25	9B6	1.609	0.093	94.76%	62	36A12	1.318	1.522	14.21%
26	9C10	1.394	3.3	-86.02%	63	36F5	1.536	1.147	35.34%
27	9C12	1.413	0.152	91.43%	64	36G1	1.331	1.008	43.18%
28	9D2	1.339	3.148	-77.45%	65	37B3	1.274	0.419	76.38%
29	9D10	1.298	2.021	-13.92%	66	37C10	1.267	1.432	19.28%
30	9E10	1.493	1.11	37.43%	67	37F2	1.334	1.365	23.06%
31	10A1	1.331	1.933	-8.96%	68	37H4	1.502	0.112	93.69%
32	10D10	1.378	1.966	-10.82%	69	38B2	1.607	1.029	42.00%
33	10E6	1.260	1.345	24.18%	70	38B6	1.522	0.814	54.11%
34	12C2	1.749	2.990	-68.55%	71	38F11	1.254	1.029	42.00%
35	12G3	1.296	0.806	54.57%	72	39D11	1.348	0.814	54.11%
36	13A1	1.549	0.245	86.19%	73	40C5	1.370	1.827	-2.99%
37	13C3	0.943	2.641	-48.87%	74	40H4	1.779	2.811	-58.46%

Table. S2. Binding capacity and neutralizing activity analysis of 17 monoclonal Abs.

mAbs	Binding to RBD		Binding to RBD_{V367F}	SARS-CoV2 Live virus	SARS-CoV2 Pseudovirus		SARS-CoV2(V367F) Pseudovirus		ELISA RBD His Blocking
	K_D (nM)	Competing w/ACE2	K_D (nM)	IC_{50} (nM)	IC_{50} (nM)	IC_{80} (nM)	IC_{50} (nM)	IC_{80} (nM)	IC_{50} (nM)
MA1	0.45	+++	0.23	0.057	0.091	0.24	0.78	1.36	3.25
MA2	0.51	+++	1.68	0.099	0.083	0.13	0.23	0.91	2.56
MA3	0.58	++	0.54	0.158	0.17	0.57	0.063	0.11	1.24
MA4	0.71	+++	0.75	n.d.	0.41	1.06	0.25	0.59	1.66
MA5	1.31	+++	4.42	0.306	1.84	9.20	3.54	5.73	3.50
MA6	0.26	+++	0.034	0.169	0.10	0.50	0.36	0.64	1.93
MA7	0.67	+++	0.87	0.198	0.47	0.35	0.042	0.10	2.53
MA8	1.24	+++	0.84	0.034	0.47	0.94	0.039	0.15	2.19
MA9	1.51	+++	6.69	0.093	n.d.	n.d.	n.d.	n.d.	5.67
MA10	1.11	++	0.75	0.088	0.11	0.34	0.084	0.14	4.10
MA11	31.2	++	19.8	n.d.	1.63	5.12	0.59	2.58	4.48
MA12	5.60	+++	3.48	n.d.	0.34	2.69	1.11	3.07	1.52
MA13	18.56	+	15.2	n.d.	n.d.	n.d.	n.d.	n.d.	11.13
MA14	19.32	+	5.78	n.d.	n.d.	n.d.	n.d.	n.d.	4.91
MA15	260	-	172	n.d.	0.41	1.18	0.67	3.01	1.70
MA16	77.0	+	26.6	n.d.	1.27	14.67	7.90	19.58	2.90
MA17	36.5	++	22.7	n.d.	n.d.	n.d.	n.d.	n.d.	3.14

mAb binding to RBD or RBD_{V367F} was presented by K_D: "+++," >80% competition with ACE2; "++," 50% to 80%; "+," 20% to 50%; and "−," <20%. IC_{50} represents the half-maximal concentration, whereas IC_{80} is the 80% inhibitory concentration in the pseudovirus neutralization assay. N.d., not done.

Table. S3. Reactivities of RBD-specific mAbs against reduced and unreduced RBDs.

mAbs	RBD-Fc	Reduced RBD-Fc
MA3	2.728	0.081
MA16	2.592	0.133
MA10	2.545	0.055
MA6	2.917	0.057
MA11	2.730	0.055
MA7	2.535	0.069
MA12	2.860	0.076
MA15	3.054	0.068
MA2	2.667	0.056
MA14	2.555	0.062
MA5	2.678	0.071
MA8	2.592	0.063
MA1	2.626	0.060
MA17	2.705	0.071
MA13	2.383	0.053
MA9	2.763	0.058
